# Spoof Detection for Finger-Vein Recognition System Using NIR Camera

**DOI:** 10.3390/s17102261

**Published:** 2017-10-01

**Authors:** Dat Tien Nguyen, Hyo Sik Yoon, Tuyen Danh Pham, Kang Ryoung Park

**Affiliations:** Division of Electronics and Electrical Engineering, Dongguk University, 30 Pildong-ro 1-gil, Jung-gu, Seoul 100-715, Korea; nguyentiendat@dongguk.edu (D.T.N.); steve10@hanmail.net (H.S.Y.); phamdanhtuyen@gmail.com (T.D.P.)

**Keywords:** NIR camera-based finger-vein recognition, spoof detection, presentation attack detection, convolutional neural network, transfer learning

## Abstract

Finger-vein recognition, a new and advanced biometrics recognition method, is attracting the attention of researchers because of its advantages such as high recognition performance and lesser likelihood of theft and inaccuracies occurring on account of skin condition defects. However, as reported by previous researchers, it is possible to attack a finger-vein recognition system by using presentation attack (fake) finger-vein images. As a result, spoof detection, named as presentation attack detection (PAD), is necessary in such recognition systems. Previous attempts to establish PAD methods primarily focused on designing feature extractors by hand (handcrafted feature extractor) based on the observations of the researchers about the difference between real (live) and presentation attack finger-vein images. Therefore, the detection performance was limited. Recently, the deep learning framework has been successfully applied in computer vision and delivered superior results compared to traditional handcrafted methods on various computer vision applications such as image-based face recognition, gender recognition and image classification. In this paper, we propose a PAD method for near-infrared (NIR) camera-based finger-vein recognition system using convolutional neural network (CNN) to enhance the detection ability of previous handcrafted methods. Using the CNN method, we can derive a more suitable feature extractor for PAD than the other handcrafted methods using a training procedure. We further process the extracted image features to enhance the presentation attack finger-vein image detection ability of the CNN method using principal component analysis method (PCA) for dimensionality reduction of feature space and support vector machine (SVM) for classification. Through extensive experimental results, we confirm that our proposed method is adequate for presentation attack finger-vein image detection and it can deliver superior detection results compared to CNN-based methods and other previous handcrafted methods.

## 1. Introduction

With the ubiquity of digital systems, applications today need enhanced security to protect sensitive user information. In some smart systems such as the immigration management system at the airport and/or the management systems in companies, the correct identification of individuals play an important role in management operations [1,2,3]. For this requirement, many traditional methods have been proposed by researchers, which can be classified into two main categories: token-based methods and knowledge-based methods [1]. However, these methods have several limitations such as inconvenience, hard to remember (complex password) and easy to be stolen. To overcome the limitations of the token-based and knowledge-based methods, biometric-based methods are increasingly being used as an alternative using the information from several physical and/or behavioral characteristics of people as the key (or password) to protect the personal information [1]. Many biometric features have been used in applications such as fingerprint [4,5], face [6,7], finger-vein [8,9,10], palm-vein [11,12] and iris [13,14,15]. The use of biometric features offers several advantages over the token-based and knowledge-based methods. Firstly, since the users’ physical features serve as the key (or password) to access their individual information resources, they do not need to carry the keys (cards) or remember the passwords. Secondly, it is difficult to steal biometric information because biometric features belong to body of each individual (fingerprint, finger-vein or iris pattern).

Although the biometric-based methods have proven efficient for authentication with high recognition rate [4,5,6,7,8,9,10,11,12,13,14,15], they still have their limitations. There are two main problems with a biometric system: the effect of image capturing condition and the potential for spoofing attacks. As proven in previous studies, the image capturing condition has a strong impact on recognition performance. For example, the performance of a face recognition system is degraded by the non-uniformity of illumination at the place where the image is being captured [16]. Similarly, the fingerprint recognition system can be affected by poor quality or resolution of fingerprint images [17], etc. Finger-vein recognition systems can also suffer due to the misalignment of input images [18]. Fortunately, these negative effects are now limited following extensive research. However, the problem of spoofing attacks persists, with various studies indicating that it is possible to attack a biometric system using presentation attack (fake) images [19,20,21,22].

Recently, finger-vein recognition has been developed and proven to be an efficient biometric authentication feature. Unlike other biometric features, the finger-vein biometric authentication procedure uses the pattern of blood vessels that is underneath the skin of the fingers to establish an individual’s identity. Therefore, skin condition has little impact on the process and it can be very difficult to steal finger-vein features because a near-infrared light (NIR) source is required to capture the blood vessel structure. However, it is still possible to spoof the finger-vein recognition system by using a stolen finger-vein image. As proven in previous research [23,24,25,26,27,28], the spoofing-attack can be done by printing the stolen finger-vein image on certain materials (such as paper or film) using carbon ink and attaching it on a real (live) finger during the image acquisition. Therefore, spoof detection methods, named as presentation attack detection (PAD) methods, for finger-vein biometric systems are necessary to protect the finger-vein recognition system from spoofing attacks.

Over time, many researchers have proposed various methods for PAD for finger-vein recognition system [23,24,25,26,27,28]. One of the earliest studies conducted by Qin et al. [28] used the dynamic information from successive images to detect the real finger-vein images. This research is based on the observation that the size of the vein pattern (blood vessels) changes minutely based on the heart rate. However, this method requires processing of successive images. Nguyen et al. [24] analyzed the finger-vein images in both frequency and spatial domain using the Fourier and wavelet transform methods. As indicated by this research, the frequency information can be used for detecting presentation attack on finger-vein images. In recent research by Tome et al. [23], several approaches were proposed for presentation attack finger-vein image detection including the use of average vertical energy of the Fourier spectrum, the use of binarized statistical image features (BSIF) and support vector machine (SVM), the use of the advantage of monogenic scale space based global descriptor, and the use of local binary pattern (LBP). Most recently, Tirunagari et al. [26] and Raghavendra et al. [27] proposed methods for presentation attack finger-vein image detection using windowed dynamic mode decomposition (DMD) and steerable pyramid feature, respectively. The researcher claimed that the steerable pyramid feature can outperform all previous research such as BSIF or LBP methods for the detection of presentation attack finger-vein images. Finally, the windowed DMD method has been proposed as an alternative method for presentation attack finger-vein image detection.

Although the aforementioned proposed methods have been demonstrated to be efficient for presentation attack finger-vein image detection, they have a limitation in terms of the feature extraction methods. In all these works, the authors designed the feature extractors according to the observation of the difference between real and presentation attack finger-vein images (handcrafted feature extractor). As a result, the extracted image features just reflect the characteristics of the real and presentation attack images in several aspects such as the difference in spatial and/or frequency domain. Therefore, the detection accuracy is limited. Recently, the learning-based method such as convolutional neural network (CNN) has been successfully applied for feature extraction for image-based recognition/classification systems and delivered superior results than traditional handcrafted feature extraction methods. Therefore, in this paper, we propose a new PAD method for finger-vein biometric system based on the convolutional neural network. Our proposed method is novel in the following four ways as compared to previous methods:

- To the best of our knowledge, this is the first approach for presentation attack detection using the deep learning framework for finger-vein biometric system. For this purpose, we apply the deep learning framework based on the CNN method for the PAD problem to overcome the limitation of previous methods that used the handcrafted methods for image feature extraction. By using a training procedure, we can learn a more suitable feature extractor for finger-vein PAD than the traditional handcrafted methods.
- Since the CNN method has a drawback of over-fitting problem caused by the huge amount of network parameters, we apply the transfer learning method instead of traditional training method for the network training procedure to minimize the over-fitting problem. By using the transfer learning method, we can utilize the optimal parameters of the existing network that were obtained using another problem. In our experiments, we used two successful CNNs, the Alex network and Visual Geometry Group (VGG) network, which were trained using ImageNet database as our preferred models for applying the transfer learning method.
- We extract image features using pre-trained CNN models to represent the input images. To reduce the effect of noise and the problem of high-dimensional features, we apply the principal component analysis (PCA) method on the extracted image features. Finally, the classification of real and presentation attack finger-vein image is done by using support vector machine (SVM).
- We collected our database of real and presentation attack finger-vein images, namely ISPR database, in which the number of images and kinds of presentation attacks are larger than those in open database. We made our database and algorithm including trained CNN model available to other researchers to compare the performance with our database and algorithm including CNN model.

In Table 1, we summarize the previous studies on the PAD for finger-vein recognition systems.

The remainder of our paper is organized as follows. In Section 2, we will provide a detailed explanation of the CNN method and propose a method for PAD based on the CNN method with transfer learning for over-fitting reduction. In Section 3, we will describe the various experiments conducted on PAD using the conventional CNN-based method as well as our method proposed in Section 2 using two different databases: ISPR database [24] and Istituto Dalle Molle di Intelligenza Artificiale Percettiva (Idiap) database [23] to demonstrate the superiority of our proposed method. Finally, we will conclude with explanations and discussions on experimental results in Section 4.

## 2. Proposed Method for PAD Based on CNN with Transfer Learning, PCA and SVM

### 2.1. Overview of the Proposed Method

**Figure 1:**
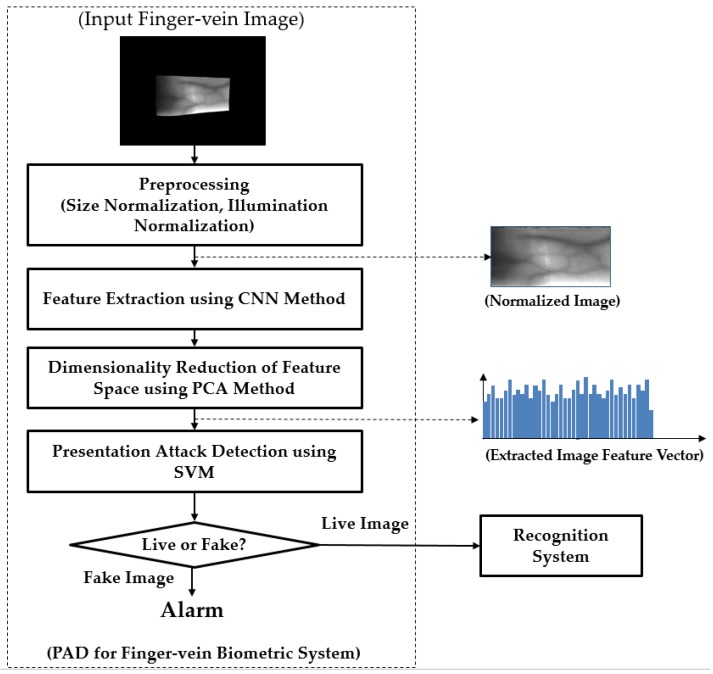
The overall structure of the proposed PAD method, and its position in finger-vein recognition systems.

Although the finger-vein recognition method has been used as an alternative for traditional methods, it is still vulnerable to attackers [23,24,25,26,27,28]. To protect the finger-vein recognition from attackers, we propose a new PAD method for the finger-vein recognition system based on feature extraction by CNN and post-processing by PCA and SVM methods for dimensionality reduction of feature space and classification, respectively. In Figure 1, we depict the flowchart of a typical finger-vein recognition system to which our proposed method can be applied. In this figure, our proposed method is depicted inside the rectangular box with the dotted lines. As shown in this figure, our proposed method is the first processing block and it is responsible for detecting the presentation attack finger-vein images before they can be input into the finger-vein recognition system. To detect the presentation attack finger-vein images, our proposed method contains several processing blocks, including the preprocessing steps, image feature extraction, feature selection, and classification, as shown in Figure 1.

Normally, the captured finger-vein images contain two parts: the vein region and the background region as shown as “Input Finger-vein Image” block in Figure 1. Therefore, we first perform a preprocessing step to extract the vein region and normalize the vein region into a rectangular region. For implementation, we used the method developed by Kang et al. [29]. As a result, we can obtain a finger-vein region image from an input captured finger-vein image. In addition to size normalization, we also apply the illumination normalization by using the zero-mean normalization to reduce the impact of the change in illumination while the finger-vein image is being captured. We term this image as “Normalized Image” for convenience, as shown in Figure 1. With this image, we perform feature extraction by using CNN-based method. Further details about CNN such as its structure and its applications will be provided in Section 2.2.

As the next step of our proposed method, we select the appropriate image features from the extracted CNN-based image features using the PCA method. The image features extracted using CNN have very high dimensions (more than 4000 components). As a result, it can increase processing time and noise for the next step of classification based on SVM. Therefore, we use the PCA method to perform the dimensionality reduction of feature space before classifying the input feature into classes of real and presentation attack finger-vein images. As a result, the dimension of extracted features is significantly reduced and the process of classifying live and fake images using SVM method becomes simpler and more efficient. The number of principal components is decided by which the best detection accuracy of our proposed method can be reached.

### 2.2. Convolutional Neural Network and Its Applications

**Figure 2:**
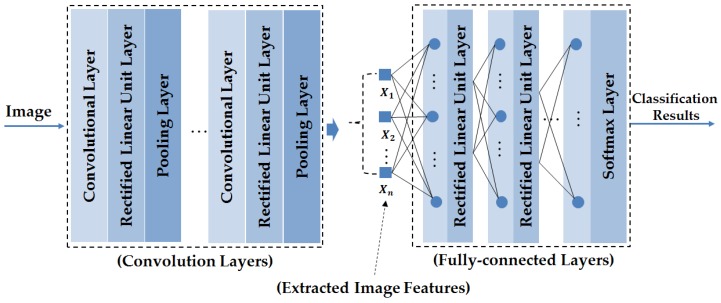
General structure of a CNN.

In the recent past, deep learning frameworks have demonstrated results that are superior to traditional methods in the field of computer vision research. For example, deep learning has been successfully applied to various image-based application systems such as face recognition [30], image classification [31,32], hand-writing digit recognition [33], person re-identification [34,35,36], gaze estimation [37], lane road detection [38], eye tracking [39] and face detection [40]. As indicated in these studies, the CNN-based deep learning method outperformed handcrafted methods by demonstrating more accurate recognition results. In Figure 2, we show the general structure of a CNN. As shown in this figure, the CNN comprises of two key parts: the convolution layers and the fully-connected layers. The convolution layers perform the image manipulation processes using the convolution operations to manipulate and extract the image features. Each convolution layer can be followed by a cross-channel normalization layer and/or a rectified linear unit (ReLU) and/or a pooling layer to transform the results of the convolution operation. As a result, we can extract an image feature vector $X=\left\{X_{1},X_{2},\ldots ,X_{n}\right\}$ as shown in Figure 2. Using this extracted image feature vector, the CNN uses a neural network (fully-connected layers in Figure 2) to classify the input image into pre-defined categories.

Although the CNN method has proven efficient for many image-based systems, it has several drawbacks. The two most significant drawbacks are the long processing time and the over-fitting problem. Due to the long processing time required, it is difficult to implement a CNN on a single general-purpose computer with limited central processing units (CPU). Fortunately, with the development of technology, this problem has been solved with the use of graphical processing unit (GPU) [31]. Using the GPUs, the CNN can be applied in real-time systems by using a large number of CPUs in parallel.

**Figure 3:**
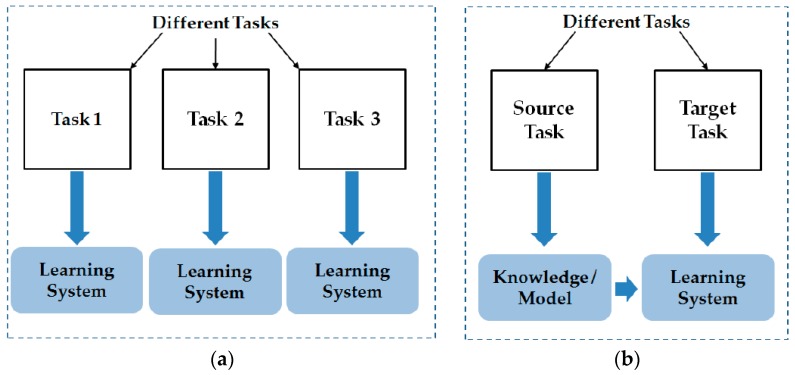
Demonstration of the difference between: (**a**) traditional machine learning technique; and (**b**) transfer learning technique.

Another problem associated with a CNN is the over-fitting problem. As described in previous studies [31,41], the CNN is constructed by learning millions of trainable parameters. Hence, the CNN-based system usually requires a huge volume of training data. Although there are several methods that have been used to reduce this problem, such as data augmentation and dropout, the amount of training data is still significant in such CNN systems. In recent years, the transfer learning method is being used to resolve this problem [42,43,44,45,46]. Using the transfer learning method, we can apply a CNN that was trained using sufficient training data for a specific problem to address a different problem [42]. This approach has been proven to be efficient for several problems, especially when large training data is scarce such as medical images [46]. In Figure 3, we show the description of the transfer learning methodology in comparison with the traditional machine learning method. As shown in this figure, the transfer learning method uses two sources to learn the system knowledge: the specific problem to be addressed (“Target Task” in Figure 3b) and the knowledge (model) obtained from another machine learning problem. In the traditional machine learning system (as depicted in Figure 3a), the system model is only learnt using the data from a single source for a given task. The use of the transfer learning method allows reusability of a CNN and transfers it to another problem. In detail, the transfer learning is defined as follows [42]:

**Definition of Transfer Learning [42]:** Given a source domain $D_{S}$ and a learning task $T_{S}$, a target domain $D_{T}$ and learning task $T_{T}$, transfer learning aims to help improve the learning of the target predictive function $f_{T}\left(.\right)$ in $D_{T}$ using the knowledge in $D_{S}$ and $T_{S}$, where $D_{S}$ ≠ $D_{T}$, or $T_{S}$ ≠ $T_{T}$.

For the experiments conducted for this study, we used two CNNs, Alex network [31] and VGG-16 network [32], for establishing the CNN architecture. For the application of the transfer learning method, the two networks are pre-trained using the ImageNet image database. The subsequent sections provide the details of these two networks: the Alex network in Section 2.2.1 and Section 2.2.2, and VGG-16 network in the Section 2.2.3 and Section 2.2.4.

To apply the transfer learning technique to these modified CNN models, we use the weights of corresponding pre-trained model to initialize the weights of the modified model. In the simplest cases of the Alex and VGG-16 architectures as described in Section 2.2.1 and Section 2.2.3, the difference between the structure of pre-trained models and corresponding modified models is not much. The difference is only at the last fully-connected layer where we replaced the number of output classes by 2. The transfer learning technique is applied by copying all the weights of a layer in the pre-trained model to the corresponding layer of the modified model. For the last fully-connected layer, the weights are randomly initialized using normal distribution of zero mean and 0.001 of standard deviation. For the customized Alex and VGG-16 architectures in Section 2.2.2 and Section 2.2.4, we carefully designed the customized models by using the same parameters as the corresponding pre-trained model structure such as filter size and stride of convolution layers. We only modified the size of input images, the number of filters in convolution layers and the number of neurons in fully-connected layers. As a result, we can use a portion of weights in pre-trained models to initialize the weights in the modified model. For more detail, we provide our code to perform transfer learning on both conventional Alex and VGG-16 networks (as described in Section 2.2.1 and Section 2.2.3) and the customized Alex and customized VGG-16 networks (as described in Section 2.2.2 and Section 2.2.4) through our laboratory website [47].

#### 2.2.1. CNN Architecture Based on Alex Network for PAD

The Alex network is one of the most popular CNNs proposed by Krizhevsky et al. [31]. This network is designed to classify images using ImageNet, a challenge that requires classifying images into 1000 different classes such as mushroom, cherry, leopard, etc. The details of the Alex network architecture used in our research are given in Table 2. In this architecture, the CNN contains five convolution layers and three fully connected layers that deliver training using two GPUs. Originally, the Alex network was used to classify the images into 1000 classes. However, for our study of PAD for finger-vein biometric system, we have only two image classes: real and presentation attack finger-vein image. Therefore, the number of neurons in the last fully-connected layer is replaced by 2 (as depicted in Table 2) instead of 1000 in the original architecture.

#### 2.2.2. Customized CNN Architecture Based on Alex Network for PAD

We observed during our study that the finger-vein images are normally not in square shape. Instead, the finger-vein images appear in rectangular shape with the width being about double the height because of the natural shape of human finger. In addition, the height of the finger-vein image is smaller than 227 pixel(s), which is used as the size of the input image in original Alex network. We can even scale the finger-vein images to the size of 227 × 227 pixels and use them as the input for the Alex network. This scheme requires a long processing time to process larger input images. In addition, since our research works on only two image classes (real and presentation attack finger-vein image) instead of 1000 classes, the use of the original Alex network requires more hardware resources. Based on this observation, we designed a new CNN structure based on the main structure of Alex network as shown in Table 3. We called this network as customized Alex network for convenience. In our design, the size of the input image is 87 × 151 pixels (height × width) instead of 227 × 227 pixels in the original Alex network. The use of this image size can reduce the processing time required by the CNN system. In addition, we use a reduced number of filters and neurons in all the layers of the networks (convolution layers and fully-connected layers). Using the lesser number of filters and number of neurons in the fully connected layer can reduce the complexity of the CNN. Consequently, the number of parameters in the network is reduced, which is beneficial for system training and testing. Using the original structure of the Alex network as depicted in Table 2, the training process must learn over 56 million network parameters using training data. However, the volume of the network parameters is reduced to about 12 million using the customized Alex network. In our experiments, we will evaluate and compare the PAD performances of both the networks (Alex network and customized Alex network).

#### 2.2.3. CNN Architecture Based on VGG Network for PAD

As demonstrated by a research by Simonyan et al. [32], the depth (the number of layers) plays an important role in the performance of a CNN-based method. In their research, they proposed two CNN architectures called VGG-16 that contains 16 layers in depth (convolution layers and fully-connected layers) and VGG-19 that contains 19 layers in depth, termed as VGG networks in our paper for convenience. These architectures are much more complex than the architecture of the Alex network described in Section 2.2.1 and Section 2.2.2. Through experiments using the ImageNet database, they proved that these CNN architectures outperform other architectures by delivering the up-to-date classification results. To investigate the performance of the PAD according to the depth of CNN, we also use the VGG network and its simpler version in our experiments. In our study, we use the VGG-16 network architecture as the reference CNN architecture. The detailed description of the VGG-16 network in our research is provided in Table 4.

#### 2.2.4. Customized CNN Architecture Based on VGG Network for PAD

Similar to our approach with the Alex network, we also customize the structure of the VGG-16 network to reduce the complexity of the network by reducing the size of the input image and the number of filters and neurons in convolution layers and fully-connected layers, respectively, while keeping the number of layers the same as the original VGG-16 network. As a result, we created a new VGG-16-based network that has lower complexity than the original VGG-16 network, and termed it as the customized VGG-16 network for convenience. The detailed description of the customized VGG-16 network is given in Table 5. In this CNN architecture, the size of the input finger-vein images is 128 × 256, which is smaller than the 224 × 224 size used in the original VGG-16 network. By using the customized VGG-16 architectures, the number of parameters in the network is reduced from over 134 million in the original VGG-16 network to approximately 23 million in the customized VGG-16 network, which helps reduce the processing time for training and testing the network. In our experiments, we perform PAD tasks using both the networks (VGG-16 and customized VGG-16 network) and compare the detection performance.

### 2.3. Image Feature Extraction and Presentation Attack Image Detection Using PCA and SVM

As described in Section 2.1, our proposed method uses a pre-trained CNN model obtained from the training process of the CNN architecture described in Section 2.2 for the image feature extraction. In contrast to handcrafted feature extraction methods used in the past such as LBP [23], BSIF [23], windowed DMD [26], pyramid decomposition [27], Fourier descriptor [23] and wavelet descriptor [24], the CNN model was obtained by a training process using a large amount of real and presentation attack finger-vein images. Therefore, the CNN model can serve as a more suitable feature extractor than the other handcrafted methods. As explained in Section 2.2, we use four CNN models with different sizes (number of filters) and depths (number of layers) based on two popular successful CNNs: Alex network [31] and VGG-16 network [32]. Using the original structures of Alex and VGG-16 networks, we can extract a feature vector of 4096-component (4096-dimensional feature vector) for each input finger-vein image using the output at the second fully-connected layer (fc7) as shown in Table 2 and Table 4. Using the same procedure, we can extract a feature vector of 1024-component using the customized CNN structures of Alex and VGG-16 networks as shown in Table 3 and Table 5. Although we can directly use these features as the inputs of SVM to classify the real and presentation attack finger-vein images, it is not the appropriate option since the dimensions of the input feature vectors are very high. The use of high-dimensional feature vectors increases the processing time of SVM and makes the SVM classifier become complex. To overcome this problem, we propose the use of the PCA method for dimensionality reduction of feature space before using the SVM for classification [48].

As the final step of our proposed method, we use the SVM for classifying the input images into two classes: real and presentation attack classes. The SVM method tries to classify the original data by transforming them into a higher dimensional space in which the data of each class is separated from the other classes using kernel functions. For our problem of real and presentation attack finger-vein image classification, the class label of test images will be identified by evaluating the sign function of Equation (1). In our experiments, we will use three different kinds of SVM kernel functions, including the linear kernel, radial basic function (RBF) and polynomial function as shown in Equations (2)–(4). In addition, we use the MATLAB environment for implementing the CNN, PCA and SVM algorithms and for measuring the performances of the detection systems [49].

(1) $$f\left(x\right)=sign(\overset{k}{\underset{i=1}{\sum }}a_{i}y_{i}K\left(x,x_{i}\right)+b)$$

(2) $$\mathrm{Linear}\;\mathrm{kernel}:\;K\left(x_{i},x_{j}\right)=x_{i}{}^{T}x_{j}$$

(3) $$\mathrm{RBF}\;\mathrm{kernel}:\;K\left(x_{i},x_{j}\right)=e^{-\gamma {\left\|x_{i}-x_{j}\right\|}^{2}}$$

(4) $$\mathrm{Polynomial}\;\mathrm{kernel}:\;K\left(x_{i},x_{j}\right)={\left(\gamma x_{i}{}^{T}x_{j}+coef\right)}^{degree}$$

## 3. Experimental Results

### 3.1. Experimental Setup

**Figure 4:**
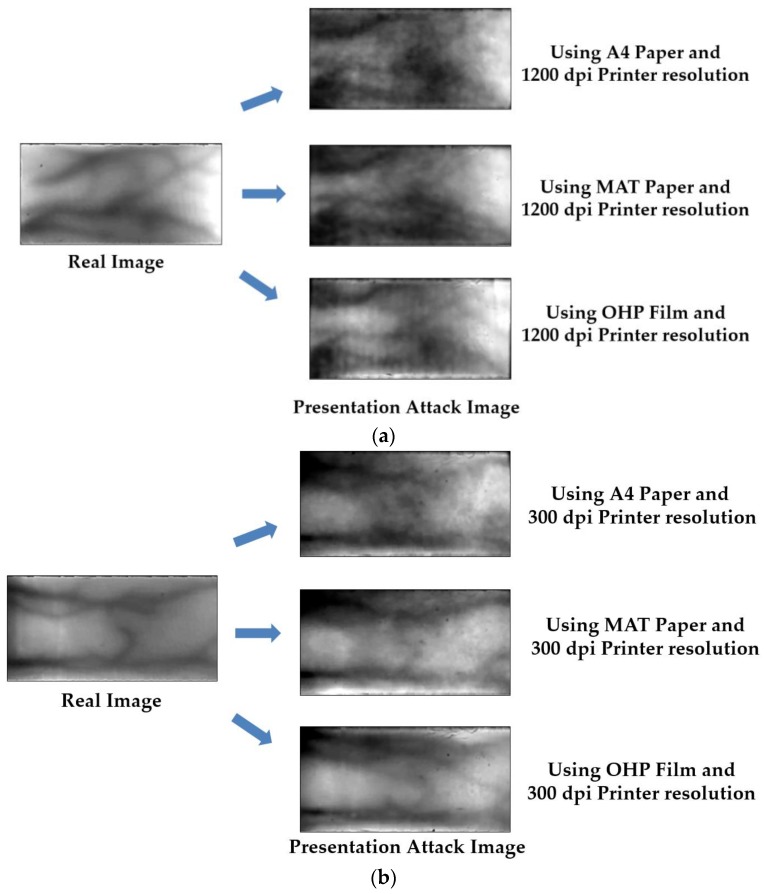
Examples of real and presentation attack finger-vein images in ISPR database: (**a**) using 1200 dpi printer resolution; and (**b**) using 300 dpi printer resolution.

To evaluate the PAD performance of our proposed method, we use two databases: ISPR database [24] and Idiap database [23]. The ISPR database consists of 3300 and 7560 images for real and presentation attack finger-vein images, respectively. The real finger-vein database was collected by capturing finger-vein images from 33 people. All 10 fingers of every individual were used and 10 trials were captured for each finger. Consequently, the real finger-vein database contains 3300 (33 people × 10 fingers × 10 trials) images. From the 3300 real finger-vein images, we selected 56 images of seven users that displayed a clear vein pattern for making the presentation attack finger-vein images. The presentation attack finger-vein image database was collected by re-capturing the printed versions of the 56 selected real finger-vein images on three different printing materials: A4 paper, MAT paper and OHP film. In addition, we used three different printing resolutions: low resolution (300 dpi), middle resolution (1200 dpi) and high resolution (2400 dpi). By using this scheme, we collected presentation attack finger-vein images that contained various characteristics specific to printing materials and printing resolution. Finally, to simulate the attack process, we captured presentation attack finger-vein images at three z-distances (the distance between the camera and the finger-vein sample) by slightly changing the z-distance during image acquisition and conducting five trials for each z-distance. As a result, a presentation attack finger-vein image database of 7560 images (56 real image × 3 printing materials × 3 printing resolutions × 3 z-distances × 5 trials) was collected. We made the ISPR database and algorithm including trained CNN model available to other researchers through the website [47] to compare the performance with this database and algorithm. In Figure 4, we show some examples of live finger-vein images and the corresponding fake finger-vein images.

In our experiments, to exploit the detection performance of our proposed method based on the kind of printing materials and printing resolution, we divided the entire ISRP database into several sub-databases according to printing materials (printed on A4 paper, printed on MAT paper, and printed on OHP film), and printing resolution (printed using 300 DPI resolution printer (Fuji Xerox DocuCentre IV C2265, Tokyo, Japan), printed using 1200 DPI resolution printer (HP LaserJet 1022, Palo Alto, CA, USA), and printed using 2400 DPI resolution printer (Samsung CLP-360 series, Seoul, South Korea)). In addition, the entire ISPR database is used for the experiment to evaluate the detection performance of our proposed method in general. For our experiments, we perform a two-fold cross-validation procedure to evaluate the performance of our proposed method. For this purpose, we divided a working database into training and testing databases twice, by which half of the real and presentation attack finger-vein images are assigned to the training database and the other half to the testing database. Using the training databases, we can learn the CNN models for image feature extraction as well as the PCA transformation matrix and SVM classifier for real and presentation attack finger-vein image classification. With these trained models of CNN, PCA and SVM, the PAD performance is measured using the testing databases. The detailed descriptions of the ISPR database as well as its sub-databases are shown in Table 6. For convenience, we named the ISPR and its sub-databases as ISPR-DB and ISPR-DB1–ISPR-DB6 as shown in Table 6.

**Figure 5:**
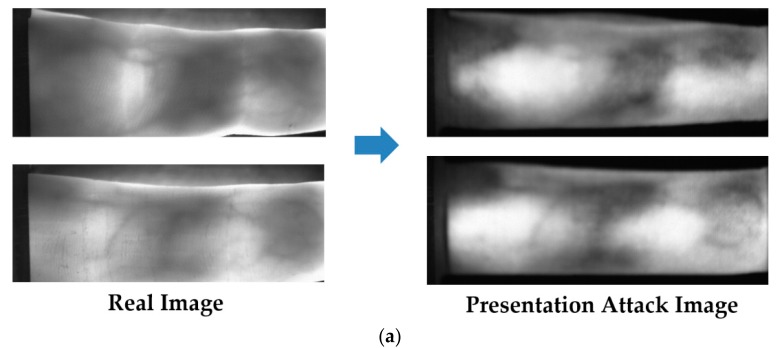

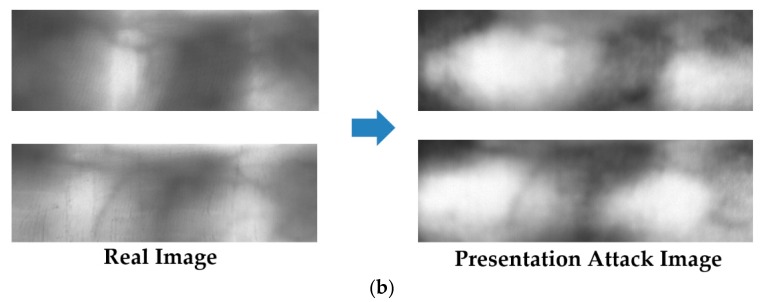
Example of real and presentation attack finger-vein images in Idiap database: (**a**) real and presentation attack finger-vein images from full image database (Idiap Full-DB); and (**b**) real and presentation attack finger-vein images from cropped image database (Idiap Cropped-DB).

The Idiap presentation attack finger-vein database (called Idiap database for convenience) is a public and famous database used for presentation attack finger-vein image detection research [23]. The Idiap database contains 440 index finger images obtained from 110 clients. From 440 real images, the authors made an additional 440 presentation attack finger-vein images by printing and recapturing method. Consequently, the Idiap database contains 880 real and presentation attack finger-vein images. For the presentation attack finger-vein image detection, the Idiap database is provided in two protocols of full image database and cropped image database. The full image database contains finger-vein images with rough boundary detection. Unlike the full image database, the cropped image database was made by localizing the finger-vein regions and removing the background regions carefully. Therefore, the images in the cropped image database contain only finger-vein regions. The use of the two protocols allows us to evaluate the effects of the background region on the performance of the detection method. In our experiment, we call the full image database as “Idiap Full-DB”, and the cropped image database as “Idiap Cropped-DB” for convenience. Figure 5 demonstrates some real and presentation attack finger-vein images in the Idiap database. In addition, the detailed description of this database with the two protocols is given in Table 7.

Based on suggestions by previous researchers [31,41], we applied two methods to reduce the over-fitting problem: dropout method and data augmentation method. For the dropout method, we applied the dropout layer in the CNN architecture, as shown in Table 2 and Table 3 for the Alex-based CNNs and Table 4 and Table 5 for the VGG-16-based CNNs. For the data augmentation approach, we artificially created the augmented database from the original training databases for both the ISPR and Idiap databases. For this purpose, we artificially made several images from each original image using shifting and cropping methods. The descriptions of the augmented databases for Idiap databases and ISPR database are given in Table 8 and Table 9, respectively. Finally, we use these augmented databases for training and evaluating the performance of our proposed method in comparison with previous methods. For the six sub-databases created from ISPR database, i.e., ISPR-DB1–ISPR-DB6, we generated 22 images from each real finger-vein image and 26 images from each presentation attack finger-vein image. For the ISPR-DB database, we generated 33 images for each real finger-vein image and 13 images for each presentation attack finger-vein image. For the Idiap database, we made 61 artificial images for each real or presentation attack finger-vein image. As shown in Table 8 and Table 9, we only perform the data augmentation on the training databases, not on testing and validation databases. Hence, the over-fitting problem is only affected by the training databases. In addition, the testing and validation databases should be retained as they were to make comparisons with previous methods on the same database. Using the data augmentation method, we can enlarge the training database and generalize the results to reduce the effect of over-fitting problem.

For a PAD system, we refer to the ISO/IEC-30107 standard (international organization for standardization (ISO) and the international electro-technical commission (IEC)) [27,50] and apply the criteria used in this standard for performance measurement of detection systems. We use two metrics for the PAD system performance measurement: the attack presentation classification error rate (APCER) and bona fide presentation classification error rate (BPCER). BPCER can as also be referred to as normal presentation classification error rate (NPCER). APCER indicates the proportion of attack presentations using the same presentation attack instrument (PAI) species incorrectly classified as bona fide presentations in the PAD subsystem in a specific scenario. BPCER indicates the proportion of bona fide presentations incorrectly classified as presentation attacks in the PAD subsystem in a specific scenario. APCER and BPCER for a given PAI are measured using Equations (5) and (6) as follows:

(5) $$\mathrm{APCER}=1-\;\left(\frac{1}{N_{PA}}\right)\overset{N_{PA}}{\underset{i=1}{\sum }}\left(RES_{i}\right)$$

(6) $$\mathrm{BPCER}=\;\frac{\sum_{i=1}^{N_{BF}}RES_{i}}{N_{BF}}$$

(7) $$\mathrm{ACER}=\;\frac{APCER+BPCER}{2}$$

In these equations, *N_PA_* indicates the number of attack presentations for the given presentation attack instrument species, *N_BF_* indicates the number of bona fide presentations, and *RES_i_* takes the value of 1 if the *i*th presentation is classified as an attack presentation and a value of 0 if it is classified as a bona fide presentation. As shown in these equations, lower values of APCER and BPCER indicate better detection performance of the PAD method. In our study, we use the average classification error rate (ACER) that is calculated using Equation (7) to measure the average error of the detection system. The Idiap database is a public database which was created by Idiap research institute in Martigny, Switzerland. In this database, the training and testing sub-databases were pre-determined by the provider of database so that images of same client (user) are only included in either training or testing dataset. For fair comparison with other researcher’s methods using this database, we followed this division scheme of training and testing sub-databases.

In our experiments with the ISPR database, we perform a two-fold cross-validation procedure for measuring the presentation detection accuracy of the detection system. In detail, in the first fold validation, we assigned the images of four users randomly selected among seven users as the training dataset, and the images of the other three users are assigned as the testing dataset. In the second fold validation, the images of the three users (used for testing in the first fold validation) with those of additional one user are used as the training dataset, and the images of the remained three users are used for testing. As a result, the images of same finger and same user are only included in either training or testing dataset. By conclusion, we performed tests on separated groups of users for the training and testing sets to properly demonstrate the generalization capability of our method.

Therefore, we measure the APCER, BPCER and ACER values for each trials of cross-validation. Finally, the presentation detection accuracies of the system (APCER, BPCER and ACER) are measured by taking the average value of the two corresponding values of the two trials. There are several differences between the images of two different databases (ISPR and Idiap databases) used in our experiments such as the capturing device, capturing environment, capturing procedure, etc. Therefore, the characteristics of images in each database are a little different as shown in Figure 4 and Figure 5. Considering this, we performed training and testing process on each database in order to ensure the detection performance.

### 3.2. Experiment Results

#### 3.2.1. PAD Accuracy Assessment Using CNN-Based Method

**Figure 6:**
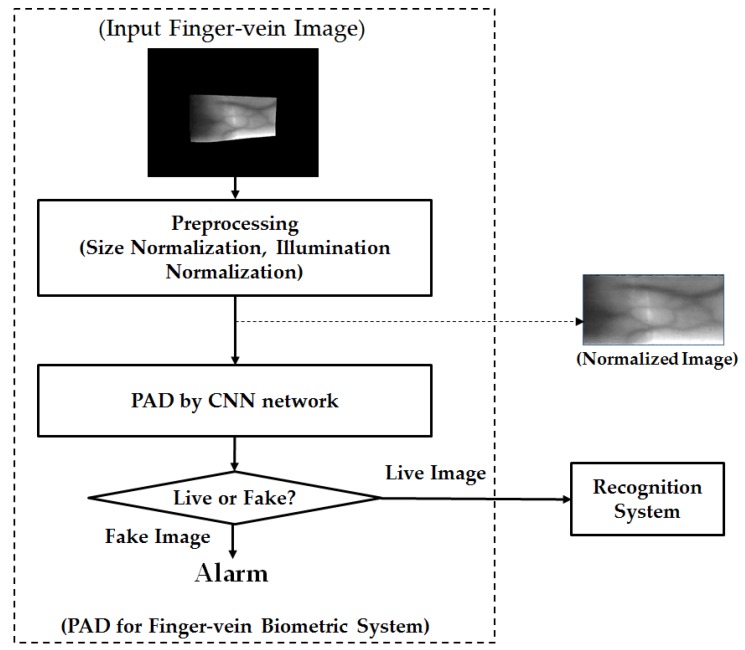
Overall procedure of CNN-based PAD method for finger-vein recognition system.

In our initial experiments, we investigated the PAD performance of systems that use only CNN-based method for classifying images into real and presentation attack classes. For this purpose, we use two CNNs including Alex network and VGG-16 network, which were described in Section 2.2.1 and Section 2.2.2, to detect the presentation attack finger-vein images directly by removing the post-processing steps by PCA and SVM shown in Figure 1. As a result, the overall procedure for the PAD method in Figure 1 is changed and the modified procedure is depicted in Figure 6. In addition, we also perform the experiments using two CNN architectures in two training modes: with and without applying the transfer learning method. For the experiments without applying the transfer learning method, the model parameters are randomly initialized using Gaussian distribution with zero-mean and a standard deviation of 0.001.

As shown in Section 3.1, we use two databases for evaluating the detection performance of PAD system: ISPR database and Idiap database. Although there are several sub-databases which were derived from ISPR database based on the printing material and printing resolution as shown in Table 6 and Table 9, we only use the entire ISPR database (ISPR-DB database) in the experiments in this section because it is the largest database that contains all other sub-databases. The other sub-databases (ISPR-DB1–ISPR-DB6) will be used in our subsequent experiments to investigate the detection performance of our proposed method based on printing materials and printing resolution. The detailed experimental results of the use of CNN architectures based on Alex network and VGG-16 network are demonstrated in Table 10 and Table 11, respectively.

In Table 10, we show the experimental results using the CNN architecture based on Alex network. Using the ISPR-DB, we obtained the APCER of 2.50% and the corresponding BPCER of 0.8073%. On an average, we obtained an ACER value of about 1.6536% when the transfer learning technique was not applied. Applying the transfer learning method on the Alex network reduced the error significantly. APCER reduced from 2.50 to 0.2018% and BPCER reduced from 0.8073 to 0.1863%. As a result, the ACER is reduced from 1.6536 to 0.194%. Similarly, when using the Idiap databases, we obtained the ACER value of 0.75% and 2.5% using Alex network without transfer learning on Idiap Full-DB and Idiap Cropped-DB databases respectively. These errors are then reduced to 0.00% using Alex network with the transfer learning method. These results demonstrate that the CNN architecture based on the Alex network was successfully used to detect the presentation attack finger-vein images. In addition, the transfer learning method outperforms the conventional CNN method using CNN architecture based on Alex network.

**Figure 7:**
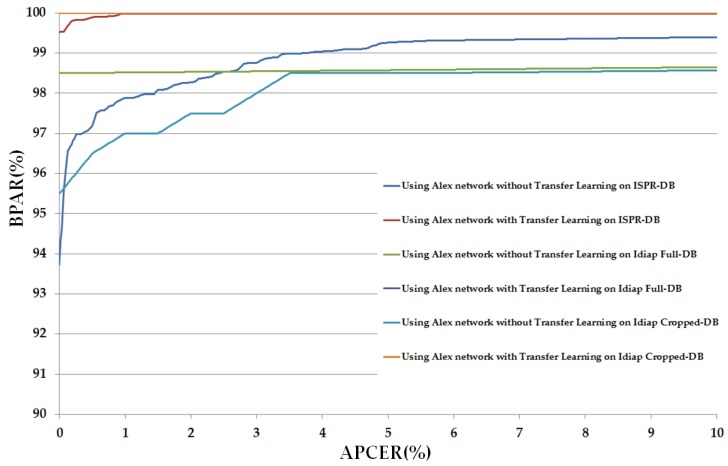
The DET curves of PAD method based on Alex network using three different databases: ISPR-DB, Idiap Full-DB and Idiap Cropped-DB database; and two training modes: with and without applying the transfer learning method.

In Figure 7, we depicted the change in APCER according to BPCER values of these above experiments. In this figure, we draw the graph of APCER versus BPAR, where BPAR indicates the bona fide acceptance rate and is calculated as: 100 (%)–BPCER (%). BPAR is defined as the proportion of the bona fide images that were correctly classified as bona fide images. We term this figure as the detection error tradeoff (DET) curve in our research. As shown in Table 10, the detection errors of the Idiap databases (Idiap Full-DB and Idiap Cropped-DB database) were reduced to zero using the transfer learning method. Therefore, DET curves of these experiments are identical, as shown in Figure 7, which indicates that transfer learning is more suitable for training and convergence of CNN than the conventional training method of CNN. In addition, this figure also demonstrates the ability of Alex network in detecting the presentation attack finger-vein images by producing small detection errors.

**Figure 8:**
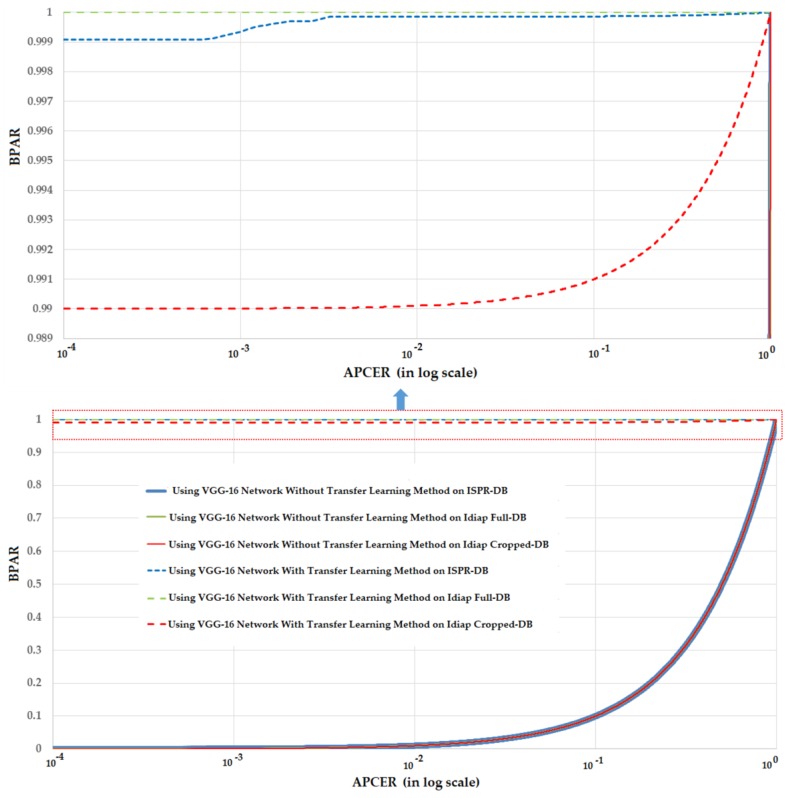
The DET curves of PAD method (in log scale) based on VGG-16 network with three different databases: ISPR-DB, Idiap Full-DB and Idiap Cropped-DB database; and two training modes: with and without applying the transfer learning method.

Similar to Table 10 and Figure 7 but using the VGG-16 network for PAD systems, Table 11 and Figure 8 show the experimental results and DET curves using ISPR-DB and Idiap databases. As shown in Table 11, the VGG-16 network works poorly on all three databases of ISPR-DB, Idiap Full-DB and Idiap Cropped-DB databases when the transfer learning method is not applied. However, using the transfer learning method on VGG-16 network, the detection accuracies became much better. The ACER of ISPR-DB is reduced from 50.00% using VGG-16 network without transfer learning method to 0.062% using VGG-16 network with transfer learning method. In the case of Idiap database, the ACER is reduced from 50.00% to 0.00% for the cases of using the Idiap Full-DB database and 0.50% for the case of using Idiap Cropped-DB database. As shown in Table 11, the detection accuracies for CNN-based method without transfer learning were 50%. As a result, the DET curves of these experiments are identical as shown in Figure 8. Through these experiments, we conclude that the CNN-based method could be sufficient for the PAD problem in finger-vein biometric system. In addition, the detection performance can be enhanced significantly using the transfer learning method in which the system parameters are manually initialized using the pre-trained parameters from another problem.

There are several reasons for the poor detection results of VGG-16 network when the transfer learning method is not applied. Firstly, as explained in Section 2.2.2, the VGG-16 network described in Table 4 contains over 134 million parameters. This large volume of parameters causes the over-fitting problem when the training database is not large enough. Secondly, as shown in Table 8 and Table 9, the volume of training data in our experiment is smaller than the size of the ImageNet database. Therefore, the training process without careful initialization of parameters is not successfully done using our databases. In contrast, applying the transfer learning method on the VGG-16 network produces sufficient accurate detection results, as shown in the right part of Table 11. This is because we used the parameters of pre-trained model as the initial parameters of the model in our problem. Consequently, the filter’s coefficients are suitable for extracting the image features and the weights in fully-connected layers are good for classification. These results again confirm the relative effectiveness of the transfer learning method on the PAD problem for finger-vein biometric system over the traditional CNN methods.

#### 3.2.2. PAD Accuracy Assessment Using Our Proposed Method Based on Alex and VGG-16 Network CNN Architectures

As a result of the experiments in Section 3.2.1, we can see that the CNN-based method is sufficient for PAD in finger-vein recognition system. However, as explained in Section 2.2, the CNNs contain a huge number of parameters. Because of this problem, the classification by using the fully-connected layers can cause over-fitting problem, which, in turn, reduces the presentation attack detection performance. In this section, we will evaluate the detection performance of our proposed method using ISPR-DB and Idiap databases (Full-DB and Cropped-DB database). As depicted in Figure 1, our proposed method performs the post-processing steps to enhance the detection accuracy of conventional CNN-based method using PCA and SVM methods. The method uses the pre-trained CNN models obtained by the experiments in Section 3.2.1 to extract the finger-vein image features. With the extracted image features, we continue performing the PCA method to reduce the dimensionality of feature space and use SVM to classify the real and presentation attack finger-vein images. As explained in Section 2.3, we used three kinds of SVM kernel for experiments: linear, RBF and polynomial.

**Figure 9:**
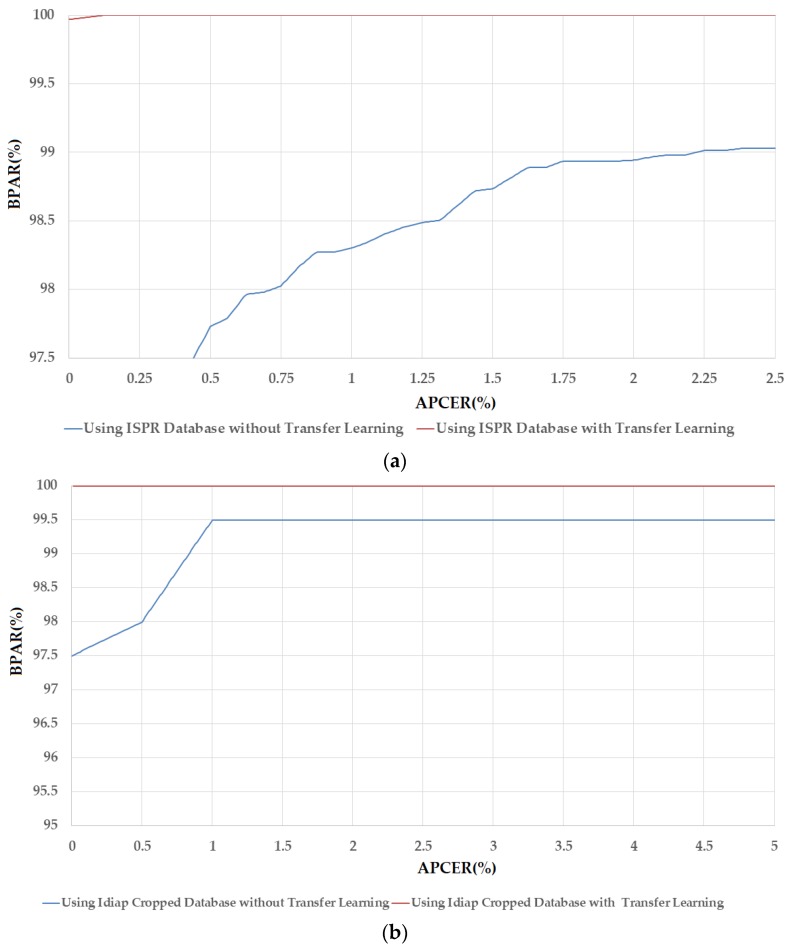
DET curves of various system configurations using our proposed method based on Alex network on two different databases of: (**a**) ISPR-DB database; and (**b**) Idiap Cropped-DB database.

In the first experiment in this section, we use the pre-trained CNN models based on the Alex network architecture to extract the image features for our proposed method. The detailed experiment results of this experiment were shown in Table 12 using two training protocols (without and with transfer learning). In addition, we also report the number of PCA coefficient corresponding to each experimental result and denoted as “No. PC” in Table 12, Table 13, Table 14, Table 15, Table 16, Table 17, Table 18, Table 19 and Table 20. In Figure 9, we show the DET curves of various system configurations corresponding to the results in Table 12. As shown in this table, using the ISPR-DB database and CNN model without transfer learning, we obtained the best detection error (ACER) of 1.1800% using polynomial kernel of SVM method. These error values are smaller than the error of 1.6536% of the system that uses only the CNN-based method without applying the transfer learning, as shown in Table 10. With transfer learning method, the error is further reduced to 0.0311% using linear kernel of SVM method. Compared to the errors in Table 10, we see that these errors are lesser than the 1.6536% error occurring as a result of using Alex network without transfer learning and also lesser than the 0.1940% using Alex network with transfer learning. These results demonstrate that our proposed method outperforms the CNN-based method for PAD using Alex network. In Figure 9a, we show the DET curves of these experiments. This figure again demonstrates the advantage of our proposed method over the conventional CNN-based method.

Similar to the experiments with ISPR-DB, we continue measuring the detection performance using Idiap databases (Full-DB and Cropped-DB database) and the results were shown in the later part of Table 12. As shown in this table, we obtained the smallest error (ACER) of 0.0% RBF kernel of SVM method on Idiap Full-DB database without applying the transfer learning method. With transfer learning method, this error is also 0.0% using RBF kernel. Because the errors of both cases are 0.0%, the DET curves of these experiments are the ideal curves (the horizontal curves at BPAR of 100%). Therefore, we do not show them in Figure 9. Finally, using the Idiap Cropped-DB database, we obtained the smallest error (ACER) of 1.0% using RBF kernel of SVM method. This result is reduced to 0.0% with the application of transfer learning method and linear kernel. The DET curves of system configuration in these experiments are shown in Figure 9b. In summary, we obtained the error of 0.00% using either Idiap Full-DB or Idiap Cropped-DB database using our proposed method and the Alex network.

**Figure 10:**
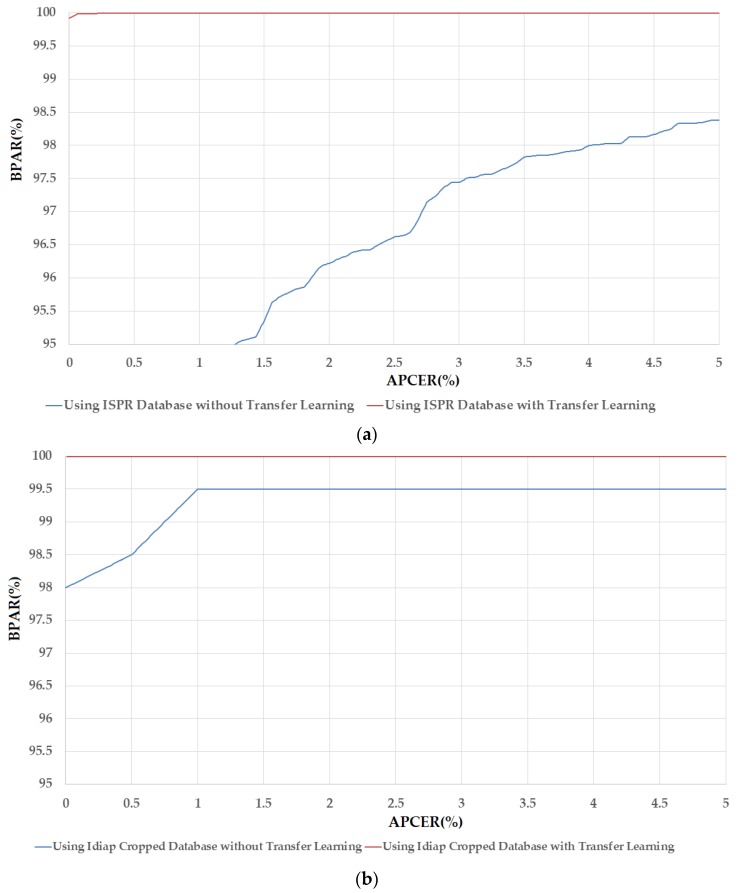
The DET curves of various system configurations using our proposed method based on VGG-16 network on two different databases: (**a**) ISPR-DB database; and (**b**) Idiap Cropped-DB database.

Similar to the above experiments, we performed our subsequent experiments using the VGG-16 network, which was described in Table 4. The detailed experimental results are shown in Table 13 and Figure 10. Using the ISPR-DB database, we obtained the smallest error (ACER) of 2.8494% using RBF kernel of SVM method, without transfer learning method. In comparison, the results depicted in Table 12, which were obtained using Alex network, are lower. However, using the transfer learning method, the error was reduced to 0.031% using linear kernel of SVM method. We can see that this error is equal to that in Table 12, which were obtained using the Alex network with the same procedure with this experiment on ISPR-DB. This phenomenon is a result of the complexity of CNN architecture. As explained in Section 2.2.1 and Section 2.2.3, the number of network parameters in the Alex network is approximately 56 million, while this number in the VGG-16 network is approximately 134 million. Therefore, without applying the transfer learning method, the over-fitting problem causes stronger effects in VGG-16 network than the Alex network. However, by using the transfer learning method, the detection performances were enhanced. This result again demonstrates the superiority of the transfer learning method over the conventional CNN method. In Figure 10a, we show the DET curves of these experiments to visually demonstrate the enhanced efficiency of the transfer learning method over the conventional CNN method.

In the last part of Table 13, we show the results of the experiments using Idiap Full-DB and Idiap Cropped-DB databases. Using the Idiap Full-DB database, we obtained the smallest errors of 0.0% in both the cases: with and without using transfer learning method. Thus, the DETs of experiments using Idiap Full-DB database are ideal curves. Therefore, we do not draw them in Figure 10. Using the Idiap Cropped-DB database, we obtained the smallest error 1.0% using RBF kernel of SVM method. This error is reduced to 0.00% using the transfer learning method. Figure 10b shows the DET curves of these experiments. Through the experiments described in Section 3.2.1 and Section 3.2.2, we conclude that the proposed method that used the CNN with transfer learning and post-processing by PCA and SVM outperformed the conventional CNN-based method for PAD for finger-vein biometric system. By comparing the PAD errors in Table 10, Table 11, Table 12 and Table 13, we can see that the VGG-16 network delivered a slight higher detection accuracy (high detection accuracy is indicated by low detection error) than the Alex network. This result confirms the effects of the depth of CNN on the detection system. In addition, it can be observed in Table 12 and Table 13 that the best detection accuracy (the smallest error value) of ISPR-DB database was 0.0311% using linear kernel of SVM method; and similarly, those of Idiap Full-DB and Cropped-DB database were 0.00% using the linear or RBF kernel of SVM method. We conclude that the linear kernel is more efficient than the other kernels (RBF and polynomial kernels) in the proposed method for PAD problem.

#### 3.2.3. PAD Accuracy Assessment Using Our Proposed Method on ISPR Database Based on Printing Resolution and Printing Materials

The experiments presented in Section 3.2.1 and Section 3.2.2 were conducted using three databases: ISPR-DB and the Idiap databases (Full-DB and Cropped-DB databases). These databases contain the largest number of finger-vein images in each database without pre-classification based on some special characteristic of presentation attack finger-vein images. Therefore, these databases are sufficient for evaluating the PAD in general. However, the detection performance can vary depending on the characteristics of presentation attack images such as printing material or printing resolution. In our subsequent experiments, we used our proposed method (CNN-based method with transfer learning and post-processing by PCA and SVM) to measure the performance of the detection method based on the methods for creating the presentation attack images. As shown in Section 3.1, the Idiap databases do not contain information of presentation attack images regarding the printing characteristics such as the printing materials or printing resolution. Therefore, the Idiap databases cannot be used for our experiment in this section. However, the ISPR-DB database was captured using different printing materials (A4, MAT, and OHP film) and printing resolution (300 dpi, 1200 dpi, and 2400 dpi). For the purpose of experiments in this section, we used the six sub-databases obtained from the ISPR-DB database (ISPR-DB1–ISPR-DB6) by manually classifying the presentation attack images into six groups according to printing materials and printing resolution, as shown in Table 6 and Table 9.

For the first experiments in this section, we used our proposed method based on the Alex network architecture to evaluate the detection performance based on the characteristics of presentation attack finger-vein images. The detailed experimental results are shown in Table 14 and Table 15 for the material-based and resolution-based presentation attack finger-vein databases, respectively. First, we measured the detection errors of the ISPR-DB1–ISPR-DB3 to investigate the effects of different printing materials on the detection performance and tabulated the results in Table 14. We obtained error values (ACER) of 0.1865%, 0.0778% and 0.3725% using ISPR-DB1, ISPR-DB2 and ISPR-B3, respectively, using CNN-based method with transfer learning. Using our proposed method, these errors are significantly reduced to 0.0389%, 0.00%, and 0.0934% using ISPR-DB1–ISPR-DB3, respectively, using the polynomial kernel of SVM method. These different errors demonstrated that the printing materials have effects on presentation attack finger-vein image detection performance. Among the three kinds of printing materials, the presentation attack finger-vein images that are made by printing on OHP film are the most difficult to detect compared to the other two kinds of printing materials, i.e., A4 paper and MAT paper.

For the subsequent experiments, we measured the detection performance for the other three sub-databases of ISPR-DB4–ISPR-DB6 to investigate the effects of printing resolution on presentation detection performance. The experimental results are shown in Table 15. Using the CNN-based method, we obtained the errors of 0.132%, 0.00% and 0.2794% using the low resolution database (ISPR-DB4), medium resolution database (ISPR-DB5), and high resolution database (ISPR-DB6), respectively. These errors are reduced to 0.0389%, 0.00% and 0.0778% using our proposed method. These results demonstrate that printing resolution can make a little impact on the PAD system.

For the second experiments in this section, we used our proposed method based on VGG-16 network instead of Alex network architecture in the above experiments. The experimental results of using different printing materials are shown in Table 16. Table 17 demonstrates the corresponding results of using different printing resolution. Compared to the similar results in Table 14 and Table 15 using Alex network, we can see that lower detection errors were obtained using our proposed method based on VGG-16 network. This result is obtained because the VGG-16 network is much deeper than Alex network. From this result, we conclude that our proposed method is efficient for PAD in various conditions for making the presentation attack finger-vein images.

#### 3.2.4. PAD Accuracy Assessment Using Our Proposed Method Based on Customized Alex and VGG-16 Networks

In the experiments in the above sections, we used the original structure of Alex network and VGG-16 network as the referenced CNN architectures in our proposed method. As shown in the experimental results above, the use of Alex network or VGG-16 network is sufficient for presentation detection problem and our proposed method that combines the CNN-based method with transfer learning and post-processing methods by PCA and SVM outperform the conventional CNN-based method. In this section, we will investigate the PAD performance of our proposed method using the two new CNN architectures including customized Alex network and customized VGG-16 network as described in Section 2.2.2 and Section 2.2.4, from which we can reach more concrete conclusions about the performance of our proposed method.

In Table 18 and Table 19, we show the experimental results using the customized Alex network (described in Table 3) and the customized VGG-16 network (described in Table 5), respectively, on three main databases: ISPR-DB, Idiap Full-DB and Idiap Cropped-DB databases. Using the customized Alex network and ISPR-DB database, we obtained the error (ACER) of 0.6601% using the CNN-based method, and the smallest error of 0.2563% using our proposed method with RBF kernel of SVM method. These results are worse than the results obtained using the original Alex network in Table 10 and Table 12 where the corresponding errors are 0.194% using only CNN-based method (with transfer learning) and 0.0311% using our proposed method. Similarly, we obtained the errors of 0.264% using CNN-based method, and the smaller error of 0.2174% using our proposed method with linear kernel of SVM method, using the customized VGG-16 network. These errors are higher than similar errors of 0.0620% and 0.0311% in Table 11 and Table 13, which were obtained using VGG-16 network on ISPR-DB database. Using the Idiap Full-DB and Idiap Cropped-DB databases, we obtained the smallest error of 0.00% that is same as those in Table 12 and Table 13 when we used Alex and VGG-16 networks. From this result, we can see that the complexity of CNN is also an important factor in the performance of PAD system.

However, as we show in Table 12 and Table 13, and Table 18 and Table 19, the difference between the errors produced by original Alex network and customized Alex network on ISPR-DB database is about 0.2252% (0.2563–0.0311%) and the difference between error produced by original VGG-16 network and customized VGG-16 network is about 0.1863% (0.2174–0.0311%). These differences are not too large. Therefore, we believe that our proposed method can produce acceptable detection accuracy even if it uses a simple CNN architecture.

For our final experiments, we measured the detection accuracy of our proposed method using the customized Alex network and customized VGG-16 network on the sub-databases of ISPR-DB database. The results of the experiment are given in Table 20. Compared to the detection errors in Table 14, Table 15, Table 16 and Table 17, we can see that the detection performances using the customized networks are also worse than those of full Alex and VGG-16 network. With the ISPR-DB1 database, we obtained the smallest error of 0.3339% using the customized Alex network and 0.2251% using the customized VGG-16 network. These errors are higher than those of 0.0389% using the original Alex network (described in Table 14 and Table 16). A similar situation is also encountered with other sub-databases of ISPR-DB database (ISPR-DB2–ISPR-DB6). However, similar to the results in Table 18 and Table 19, the differences between the results from the customized network and the original network are not too significant. For example, for the ISPR-DB1 database, the difference in errors is about 0.295% (0.3339–0.0389%) and 0.1862% (0.2251–0.0389%) using the customized Alex network and the customized VGG-16 network respectively. Therefore, we again establish that our proposed method can produce acceptable detection accuracy even if it uses a simple CNN structure.

#### 3.2.5. Comparison with Various Methods

The ISPR and Idiap databases used in our experiments have been used by previous studies on the PAD problem. To demonstrate the superiority of our proposed method over the various studies, we summarized the PAD accuracies of our proposed method in comparison with those produced by previous studies. In Table 21, we show the comparison of our proposed method with previous methods adopted by Nguyen et al. [24] as well as the conventional CNN-based methods. As shown in this table, our proposed method outperforms all previous methods using the ISPR-DB database and its sub-databases of presentation attack based on printer material and resolution by producing the smallest error (ACERs) values.

Similar to Table 21, Table 22 shows the comparison of PAD errors using our proposed method with those of previous studies using Idiap Full-DB and Idiap Cropped-DB databases. The Idiap database was fully investigated in the research by Tome et al. [23]. Four methods were applied for PAD using the Idiap database: baseline method, GUC method, B-Lab method, and GRIP-PRIAMUS method. As shown in Table 22, our proposed method is comparable to the GRIP-PRIAMUS method using both the Idiap Full-DB and Idiap Cropped-DB databases. Compared to the other three methods, our proposed method delivered better detection results. Our proposed method produced a detection error (ACER) of 0.00% for both Idiap Full-DB and Idiap Cropped-DB databases. On the other hand, the baseline method produced an error of 0.00% using Idiap Full-DB database and 20.50% using the Idiap Cropped-DB database; the GUC method produced an error of 4.00% using the Idiap Full-DB database and 2.75% using Idiap Cropped-DB database. Finally, the B-Lab method produced an error of 0.00% using the Idiap Full-DB database and 1.25% using the Idiap Cropped-DB database. In addition, we compared the detection accuracy of our proposed method with the conventional CNN-based methods. As shown in this table, the detection accuracy of our proposed method is comparable with other conventional CNN-based detection method using the Idiap database. However, from the result of both Table 21 and Table 22, we can conclude that our proposed method outperforms the conventional CNN-based method by producing smaller error than those methods.

We also gave the comparison of the system performance with and without applying the PCA method in order to demonstrate the efficiency of dimensionality reduction of feature space (using PCA method) in our proposed method in Table 21 and Table 22. The case without PCA and with SVM could not be compared in our experiment. That is because the dimension of input data (obtained by CNN) to SVM is so large (as 4096) considering the huge number of training data that SVM is not operated without the dimensionality reduction of feature space by PCA method. Therefore, we compared the case without the dimensionality reduction of feature space by PCA and classification by SVM to our proposed method (with the dimensionality reduction of feature space by PCA and classification by SVM) in Table 21 and Table 22. Experimental results showed that our proposed method outperforms the other methods.

#### 3.2.6. Application of our Proposed Method on PAD for Palm-Vein Recognition Systems

For the vein-based biometrics systems, there is another recognition method that has been used for identification/recognition, called palm-vein recognition method. This recognition method is similar to finger-vein recognition by the use of vein structure inside a hand for recognition task [11,12,51,52]. Similar to finger-vein recognition systems, the palm-vein recognition systems are also vulnerable to attackers [51]. Therefore, in this section, we applied our proposed method to detect the presentation attacks for such palm-vein recognition systems. For this purpose, we used a public database, called the Idiap VERA spoofing palm-vein database [51], for our experiments. We refer to this database as “Idiap PVD” for convenience. The Idiap PVD database contains palm-vein and presentation attack images from 50 users. For each user, they captured both the left and right hand in two sessions (Sessions 1 and 2) to produce 10 images for each hand. Totally, the Idiap PVD database contains 1000 real and 1000 presentation attack images (50 persons × 2 hands × 2 Sessions × 5 trials). In addition, two protocols of full and cropped images are provided that is similar to Idiap spoofing finger-vein database in [23]. In these experiments, we again performed two-fold cross-validation procedure to measure the detection performance. In the first fold validation, we used images from 25 persons for training, and data from the other 25 persons for testing. In the second fold validation, we exchanged these two datasets of training and testing each other, and repeated the experiment. Similar to the experiments in above sections, we also performed data augmentation procedure on training databases to generalize the training databases and reduce the over-fitting problem of CNN-based methods. For this purpose, we artificially made 49 images from each original palm-vein image (real and presentation attack image) by shifting and cropping the original image in both horizontal and vertical directions. As a result, we obtained 24,500 images for training database and 500 images for testing database for our experiments. In Table 23, we show the detailed description of the Idiap PVD and its corresponding training and testing sub-databases using two-fold cross-validation scheme.

For comparison purpose, we performed experiments using four detection methods including the previous method by Nguyen et al. [24], CNN-based method using Alex network; the CNN-based method using VGG-16 network; and our proposed method using VGG-16 network. The detailed experimental results are given in Table 24. As shown in this Table, our proposed method outperforms the other methods by accurately detecting the spoofing images. From these results, we can conclude that our proposed method is not only suitable for detecting spoofing images for finger-vein recognition system, but also for the palm-vein recognition system. In addition, our proposed method outperforms the conventional CNN-based method and previous method in [24].

## 4. Conclusions

In this paper, we proposed a PAD method for finger-vein recognition systems. Our proposed method is based on the convolutional neural network with the use of transfer learning to reduce the effects of the over-fitting problem normally caused by a small amount of training data and/or the complexity of the CNN architecture. To enhance the detection performance of conventional CNN-based methods, we applied post-processing steps based on PCA method for dimensionality reduction of feature space and SVM for classification. As shown in our experimental results using ISPR database and Idiap database, the proposed method outperformed methods used previously to resolve the same problem using the same databases. In addition, the VGG network (VGG-16 network), which is much deeper than the Alex network, delivered slightly better detection performance compared to the others. We obtained the smallest error of 0.0311% using ISPR database that is much smaller than the error produced by previous research. Using the Idiap database, we obtained errors of 0.00% with both protocols of full and cropped images. Through these experimental results, we confirm the efficiency of the transfer learning method in solving the over-fitting problem of CNN caused by small amount of training data and/or the high complexity of CNN. In addition, we confirm that the CNN-based method is suitable for detection of presentation attack for finger-vein recognition system.

In future work, we plan to collect more reasonable image data of real and presentation attack to simulate all the possible cases of the PAD problem. With the new data, we will investigate the problem in more detail to make our system invariant to various possible presentation attack methods.

## Figures and Tables

**Table 1:** A summary of previous studies on presentation attack detection (PAD) methods and our proposed method.

Categories	Methods	Strength	Weakness	Accuracy (Database)
PAD methods using handcrafted methods	Uses a series of images to check the variation of vein patterns based on heart rate [28]	Easy to implement	Requires high processing time for capturing and processing successive images	Not available
	Uses the combination of features in both spatial and frequency domain through Fourier and wavelet transform [24]	Uses information from both spatial and frequency domain for fake image detection. Fair detection accuracy	More complex than the method in [28]. Detection accuracy is limited due to the uses of handcrafted image feature extractor.	Equal error rate (EER) = 2.874% (Using ISPR Database)
	Uses average vertical energy of the Fourier spectrum; BSIF feature; monogenic scale space based global descriptor; and local binary pattern on residual image [23].	Fair detection accuracy	Detection accuracy is limited due to the use of handcrafted image feature extractor.	Half total error rate (HTER) = 0.00% (Using Idiap Database)
	Uses the windowed DMD as micro-texture descriptor for fake finger-vein image detection [26]	Results comparable to previous methods		EER = 1.59% (Idiap Cropped Database) EER = 0.08% (Using Idiap Full Database)
	Uses steerable pyramids decomposition for image feature extraction [27]	Improved detection accuracy compared to some other previous proposed methods.		Average classification error rate (ACER) = about 3.0% (A collected database consists of 300 unique finger-vein instance)
PAD method using learning-based method (**Proposed Method**)	Uses CNN to learn the suitable image feature extractor Post-processing by PCA and SVM to enhance the detection performance	Suitable image feature extractor is obtained using CNN-based method. Produces very high detection accuracy compared to previous methods.	Requires a large amount of computation operations and is more complex than previous methods.	HTER = 0.00% (Using Idiap database) HTER = 0.031% (Using ISPR database)

**Table 2:** Description of CNN structure based on Alex network for PAD problem.

Layer Name	Number of Filters	Filter Size	Stride Size	Padding Size	Dropout Value	Output Size
Input Layer	n/a	n/a	n/a	n/a	n/a	227 × 227 × 3
Convolution Layer 1 (conv1)	96	11 × 11 × 3	4 × 4	0	n/a	55 × 55 × 96
Rectified Linear Unit (relu1)	n/a	n/a	n/a	n/a	n/a	55 × 55 × 96
Normalization Layer (norm1)	n/a	n/a	n/a	n/a	n/a	55 × 55 × 96
MAX Pooling Layer 1 (pool1)	1	3 × 3	2 × 2	0	n/a	27 × 27 × 96
Convolution Layer 2 (conv2)	256	5 × 5 × 48	1 × 1	2 × 2	n/a	27 × 27 × 256
Rectified Linear Unit (relu2)	n/a	n/a	n/a	n/a	n/a	27 × 27 × 256
Normalization Layer (norm2)	n/a	n/a	n/a	n/a	n/a	27 × 27 × 256
MAX Pooling Layer 2 (pool2)	1	3 × 3	2 × 2	0	n/a	13 × 13 × 256
Convolution Layer 3 (conv3)	384	3 × 3 × 256	1 × 1	1 × 1	n/a	13 × 13× 384
Rectified Linear Unit (relu3)	n/a	n/a	n/a	n/a	n/a	13 × 13 × 384
Convolution Layer 4 (conv4)	384	3 × 3 × 192	1 × 1	1 × 1	n/a	13 × 13 × 384
Rectified Linear Unit (relu4)	n/a	n/a	n/a	n/a	n/a	13 × 13 × 384
Convolution Layer 5 (conv5)	256	3 × 3 × 192	1 × 1	1 × 1	n/a	13 × 13 × 256
Rectified Linear Unit (relu5)	n/a	n/a	n/a	n/a	n/a	13 × 13 × 256
MAX Pooling Layer 5 (pool5)	1	3 × 3	2 × 2	0	n/a	6 × 6 × 256
Fully Connected Layer 1 (fc6)	n/a	n/a	n/a	n/a	n/a	4096
Dropout Layer (drop6)	n/a	n/a	n/a	n/a	0.50	4096
Rectified Linear Unit (relu6)	n/a	n/a	n/a	n/a	n/a	4096
Fully Connected Layer 2 (fc7)	n/a	n/a	n/a	n/a	n/a	4096
Rectified Linear Unit (relu7)	n/a	n/a	n/a	n/a	n/a	4096
Dropout Layer (drop7)	n/a	n/a	n/a	n/a	0.50	4096
Output Layer (fc8)	n/a	n/a	n/a	n/a	n/a	2
Softmax Layer (prob)	n/a	n/a	n/a	n/a	n/a	2
Classification Layer (output)	n/a	n/a	n/a	n/a	n/a	2

**Table 3:** Description of customized Alex network for PAD problem.

Layer Name	Number of Filters	Filter Size	Stride Size	Padding Size	Dropout Value	Output Size
Input Layer	n/a	n/a	n/a	n/a	n/a	87 × 151 × 3
Convolution Layer 1 (conv1)	96	11 × 11 × 3	2 × 2	0	n/a	39 × 71 × 96
Rectified Linear Unit (relu1)	n/a	n/a	n/a	n/a	n/a	39 × 71 × 96
Normalization Layer (norm1)	n/a	n/a	n/a	n/a	n/a	39 × 71 × 96
MAX Pooling Layer 1 (pool1)	1	3 × 3	2 × 2	0	n/a	19 × 35 × 96
Convolution Layer 2 (conv2)	128	5 × 5 × 96	1 × 1	2 × 2	n/a	19 × 35 × 128
Rectified Linear Unit (relu2)	n/a	n/a	n/a	n/a	n/a	19 × 35 × 128
Normalization Layer (norm2)	n/a	n/a	n/a	n/a	n/a	19 × 35 × 128
MAX Pooling Layer 2 (pool2)	1	3 × 3	2 × 2	0	n/a	9 × 17 × 128
Convolution Layer 3 (conv3)	192	3 × 3 × 128	1 × 1	1 × 1	n/a	9 × 17 × 192
Rectified Linear Unit (relu3)	n/a	n/a	n/a	n/a	n/a	9 × 17 × 192
Convolution Layer 4 (conv4)	192	3 × 3 × 192	1 × 1	1 × 1	n/a	9 × 17 × 192
Rectified Linear Unit (relu4)	n/a	n/a	n/a	n/a	n/a	9 × 17 × 192
Convolution Layer 5 (conv5)	128	3 × 3 × 192	1 × 1	1 × 1	n/a	9 × 17 × 128
Rectified Linear Unit (relu5)	n/a	n/a	n/a	n/a	n/a	9 × 17 × 128
MAX Pooling Layer 5 (pool5)	1	3 × 3	2 × 2	0	n/a	4 × 8 × 128
Fully Connected Layer 1 (fc6)	n/a	n/a	n/a	n/a	n/a	2048
Dropout Layer (drop6)	n/a	n/a	n/a	n/a	0.50	2048
Rectified Linear Unit (relu6)	n/a	n/a	n/a	n/a	n/a	2048
Fully Connected Layer 2 (fc7)	n/a	n/a	n/a	n/a	n/a	1024
Rectified Linear Unit (relu7)	n/a	n/a	n/a	n/a	n/a	1024
Dropout Layer (drop7)	n/a	n/a	n/a	n/a	0.50	1024
Output Layer (fc8)	n/a	n/a	n/a	n/a	n/a	2
Softmax Layer (prob)	n/a	n/a	n/a	n/a	n/a	2
Classification Layer (output)	n/a	n/a	n/a	n/a	n/a	2

**Table 4:** Description of CNN structure based on VGG-16 network for PAD problem.

Layer Name	Number of Filters	Filter Size	Stride Size	Padding Size	Dropout Value	Output Size
Input Layer	n/a	n/a	n/a	n/a	n/a	224 × 224 × 3
Convolution Layer (conv1_1)	64	3 × 3 × 3	1 × 1	1 × 1	n/a	224 × 224 × 64
Rectified Linear Unit 1 (relu1_1)	n/a	n/a	n/a	n/a	n/a	224 × 224 × 64
Convolution Layer (conv1_2)	64	3 × 3 × 64	1 × 1	1 × 1	n/a	224 × 224 × 64
Rectified Linear Unit (relu1_2)	n/a	n/a	n/a	n/a	n/a	224 × 224 × 64
MAX Pooling Layer (pool1)	1	2 × 2	2 × 2	0	n/a	112 × 112 × 64
Convolution Layer (conv2-1)	128	3 × 3 × 64	1 × 1	1 × 1	n/a	112 × 112 × 128
Rectified Linear Unit (relu2_1)	n/a	n/a	n/a	n/a	n/a	112 × 112 × 128
Convolution Layer (conv2_2)	128	3 × 3 × 128	1 × 1	1 × 1	n/a	112 × 112 × 128
Rectified Linear Unit (relu2_2)	n/a	n/a	n/a	n/a	n/a	112 × 112 × 128
MAX Pooling Layer (pool2)	1	2 × 2	2 × 2	0	n/a	56 × 56 × 128
Convolution Layer (conv3_1)	256	3 × 3 × 128	1 × 1	1 × 1	n/a	56 × 56 × 256
Rectified Linear Unit (relu3_1)	n/a	n/a	n/a	n/a	n/a	56 × 56 × 256
Convolution Layer (conv3_2)	256	3 × 3 × 256	1 × 1	1 × 1	n/a	56 × 56 × 256
Rectified Linear Unit (relu3_2)	n/a	n/a	n/a	n/a	n/a	56 × 56 × 256
Convolution Layer (conv3_3)	256	3 × 3 × 256	1 × 1	1 × 1	n/a	56 × 56 × 256
Rectified Linear Unit (relu3_3)	n/a	n/a	n/a	n/a	n/a	56 × 56 × 256
MAX Pooling Layer (pool3)	1	2 × 2	2 × 2	0	n/a	28 × 28 × 256
Convolution Layer (conv4_1)	512	3 × 3 × 256	1 × 1	1 × 1	n/a	28 × 28 × 512
Rectified Linear Unit (relu4_1)	n/a	n/a	n/a	n/a	n/a	28 × 28 × 512
Convolution Layer (conv4_2)	512	3 × 3 × 512	1 × 1	1 × 1	n/a	28 × 28 × 512
Rectified Linear Unit (relu4_2)	n/a	n/a	n/a	n/a	n/a	28 × 28 × 512
Convolution Layer (conv4_3)	512	3 × 3 × 512	1 × 1	1 × 1	n/a	28 × 28 × 512
Rectified Linear Unit (relu4_3)	n/a	n/a	n/a	n/a	n/a	28 × 28 × 512
MAX Pooling Layer (pool4)	1	2 × 2	2 × 2	0	n/a	14 × 14 × 512
Convolution Layer (conv5_1)	512	3 × 3 × 512	1 × 1	1 × 1	n/a	14 × 14 × 512
Rectified Linear Unit (relu5_1)	n/a	n/a	n/a	n/a	n/a	14 × 14 × 512
Convolution Layer (conv5_2)	512	3 × 3 × 512	1 × 1	1 × 1	n/a	14 × 14 × 512
Rectified Linear Unit (relu5_2)	n/a	n/a	n/a	n/a	n/a	14 × 14 × 512
Convolution Layer (conv5_3)	512	3 × 3 × 512	1 × 1	1 × 1	n/a	14 × 14 × 512
Rectified Linear Unit (relu5_3)	n/a	n/a	n/a	n/a	n/a	14 × 14 × 512
MAX Pooling Layer (pool5)	1	2 × 2	2 × 2	0	n/a	7 × 7 × 512
Fully Connected Layer (fc6)	n/a	n/a	n/a	n/a	n/a	4096
Rectified Linear Unit (relu6)	n/a	n/a	n/a	n/a	n/a	4096
Dropout Layer (drop6)	n/a	n/a	n/a	n/a	0.50	4096
Fully Connected Layer (fc7)	n/a	n/a	n/a	n/a	n/a	4096
Rectified Linear Unit (relu7)	n/a	n/a	n/a	n/a	n/a	4096
Dropout Layer (drop7)	n/a	n/a	n/a	n/a	0.50	4096
Output Layer (fc8)	n/a	n/a	n/a	n/a	n/a	2
Softmax Layer (prob)	n/a	n/a	n/a	n/a	n/a	2
Classification Layer (output)	n/a	n/a	n/a	n/a	n/a	2

**Table 5:** Description of customized VGG-16 network for PAD problem.

Layer Name	Number of Filters	Filter Size	Stride Size	Padding Size	Dropout Value	Output Size
Input Layer	n/a	n/a	n/a	n/a	n/a	128 × 256 × 3
Convolution Layer (conv1_1)	32	3 × 3 × 3	1 × 1	1 × 1	n/a	128 × 256 × 32
Rectified Linear Unit 1 (relu1_1)	n/a	n/a	n/a	n/a	n/a	128 × 256 × 32
Convolution Layer (conv1_2)	32	3 × 3 × 32	1 × 1	1 × 1	n/a	128 × 256 × 32
Rectified Linear Unit (relu1_2)	n/a	n/a	n/a	n/a	n/a	128 × 256 × 32
MAX Pooling Layer (pool1)	1	2 × 2	2 × 2	0	n/a	64 × 128 × 32
Convolution Layer (conv2-1)	64	3 × 3 × 32	1 × 1	1 × 1	n/a	64 × 128 × 64
Rectified Linear Unit (relu2_1)	n/a	n/a	n/a	n/a	n/a	64 × 128 × 64
Convolution Layer (conv2_2)	64	3 × 3 × 64	1 × 1	1 × 1	n/a	64 × 128 × 64
Rectified Linear Unit (relu2_2)	n/a	n/a	n/a	n/a	n/a	64 × 128 × 64
MAX Pooling Layer (pool2)	1	2 × 2	2 × 2	0	n/a	32 × 64 × 64
Convolution Layer (conv3_1)	128	3 × 3 × 64	1 × 1	1 × 1	n/a	32 × 64 × 128
Rectified Linear Unit (relu3_1)	n/a	n/a	n/a	n/a	n/a	32 × 64 × 128
Convolution Layer (conv3_2)	128	3 × 3 × 128	1 × 1	1 × 1	n/a	32 × 64 × 128
Rectified Linear Unit (relu3_2)	n/a	n/a	n/a	n/a	n/a	32 × 64 × 128
Convolution Layer (conv3_3)	128	3 × 3 × 128	1 × 1	1 × 1	n/a	32 × 64 × 128
Rectified Linear Unit (relu3_3)	n/a	n/a	n/a	n/a	n/a	32 × 64 × 128
MAX Pooling Layer (pool3)	1	2 × 2	2 × 2	0	n/a	16 × 32 × 128
Convolution Layer (conv4_1)	256	3 × 3 × 128	1 × 1	1 × 1	n/a	16 × 32 × 256
Rectified Linear Unit (relu4_1)	n/a	n/a	n/a	n/a	n/a	16 × 32 × 256
Convolution Layer (conv4_2)	256	3 × 3 × 256	1 × 1	1 × 1	n/a	16 × 32 × 256
Rectified Linear Unit (relu4_2)	n/a	n/a	n/a	n/a	n/a	16 × 32 × 256
Convolution Layer (conv4_3)	256	3 × 3 × 256	1 × 1	1 × 1	n/a	16 × 32 × 256
Rectified Linear Unit (relu4_3)	n/a	n/a	n/a	n/a	n/a	16 × 32 × 256
MAX Pooling Layer (pool4)	1	2 × 2	2 × 2	0	n/a	8 × 16 × 256
Convolution Layer (conv5_1)	256	3 × 3 × 256	1 × 1	1 × 1	n/a	8 × 16 × 256
Rectified Linear Unit (relu5_1)	n/a	n/a	n/a	n/a	n/a	8 × 16 × 256
Convolution Layer (conv5_2)	256	3 × 3 × 256	1 × 1	1 × 1	n/a	8 × 16 × 256
Rectified Linear Unit (relu5_2)	n/a	n/a	n/a	n/a	n/a	8 × 16 × 256
Convolution Layer (conv5_3)	256	3 × 3 × 256	1 × 1	1 × 1	n/a	8 × 16 × 256
Rectified Linear Unit (relu5_3)	n/a	n/a	n/a	n/a	n/a	8 × 16 × 256
MAX Pooling Layer (pool5)	1	2 × 2	2 × 2	0	n/a	4 × 8 × 256
Fully Connected Layer (fc6)	n/a	n/a	n/a	n/a	n/a	2048
Rectified Linear Unit (relu6)	n/a	n/a	n/a	n/a	n/a	2048
Dropout Layer (drop6)	n/a	n/a	n/a	n/a	0.50	2048
Fully Connected Layer (fc7)	n/a	n/a	n/a	n/a	n/a	1024
Rectified Linear Unit (relu7)	n/a	n/a	n/a	n/a	n/a	1024
Dropout Layer (drop7)	n/a	n/a	n/a	n/a	0.50	1024
Output Layer (fc8)	n/a	n/a	n/a	n/a	n/a	2
Softmax Layer (prob)	n/a	n/a	n/a	n/a	n/a	2
Classification Layer (output)	n/a	n/a	n/a	n/a	n/a	2

**Table 6:** Description of ISPR presentation attack finger-vein image database.

Image Making Protocol		Real Access			Presentation Attack Access		
		Train Set	Test Set	Total	Train Set	Test Set	Total
Material	Printed on A4 Paper (ISPR-DB1)	1700	1600	3300	1440	1080	2520
	Printed on MAT Paper (ISPR-DB2)	1700	1600	3300	1440	1080	2520
	Printed on OHP Film (ISPR-DB3)	1700	1600	3300	1440	1080	2520
Printer Resolution	Printed Using 300 DPI Resolution Printer (ISPR-DB4)	1700	1600	3300	1440	1080	2520
	Printed Using 1200 DPI Resolution Printer (ISPR-DB5)	1700	1600	3300	1440	1080	2520
	Printed Using 2400 DPI Resolution Printer (ISPR-DB6)	1700	1600	3300	1440	1080	2520
Entire Database (ISPR-DB)		1700	1600	3300	4320	3120	7560

**Table 7:** Description of and Istituto Dalle Molle di Intelligenza Artificiale Percettiva (Idiap) presentation attack finger-vein image database with two protocols of full and cropped images.

Image Making Protocol	Real Access			Presentation Attack Access		
	Train Set	Test Set	Validation Set	Train Set	Test Set	Validation Set
Full Image Database (Idiap Full-DB)	120	200	120	120	200	120
Cropped Image Database (Idiap Cropped-DB)	120	200	120	120	200	120

**Table 8:** Description of the augmented database derived from Idiap databases.

Database	Real Access			Presentation Attack Access		
	Train Set	Test Set	Validation Set	Train Set	Test Set	Validation Set
Idiap Full-DB	7440 (120 × 62)	200	120	7440 (120 × 62)	200	120
Idiap Cropped-DB	7440 (120 × 62)	200	120	7440 (120 × 62)	200	120

**Table 9:** Description of the augmented database derived from ISPR database and its sub-databases (ISPR-DB1–ISPR-DB6).

Database		Real Access		Presentation Attack Access	
		Train Set	Test Set	Train Set	Test Set
Material	Printed on A4 Paper (ISPR-DB1)	37,400 (1700 × 22)	1600	37,440 (1440 × 26)	1080
	Printed on MAT Paper (ISPR-DB2)	37,400 (1700 × 22)	1600	37,440 (1440 × 26)	1080
	Printed on OHP Film (ISPR-DB3)	37,400 (1700 × 22)	1600	37,440 (1440 × 26)	1080
Printer Resolution	Printed Using 300 DPI Resolution Printer (ISPR-DB4)	37,400 (1700 × 22)	1600	37,440 (1440 × 26)	1080
	Printed Using 1200 DPI Resolution Printer (ISPR-DB5)	37,400 (1700 × 22)	1600	37,440 (1440 × 26)	1080
	Printed Using 2400 DPI Resolution Printer (ISPR-DB6)	37,400 (1700 × 22)	1600	37,440 (1440 × 26)	1080
Entire Database (ISPR-DB)		56,100 (1700 × 33)	1600	56,160 (4320 × 13)	3240

**Table 10:** PAD errors of CNN-based method with and without applying the transfer learning technique using Alex network architecture depicted in Table 2 (unit: %).

Database	Without Transfer Learning			With Transfer Learning		
	APCER	BPCER	ACER	APCER	BPCER	ACER
ISPR-DB	2.5000	0.8073	1.6536	0.2018	0.1863	**0.1940**
Idiap Full-DB	0.000	1.5000	0.7500	0.0000	0.0000	**0.0000**
Idiap Cropped-DB	2.5000	2.5000	2.500	0.0000	0.0000	**0.0000**

**Table 11:** PAD errors of CNN-based method with and without applying the transfer learning technique using VGG-16 network architecture in Table 4 (unit: %).

Database	Without Transfer Learning			With Transfer Learning		
	APCER	BPCER	ACER	APCER	BPCER	ACER
ISPR-DB	0.0000	100.00	50.00	0.0000	0.1240	**0.0620**
Idiap (Full-DB)	0.0000	100.00	50.00	0.0000	0.0000	**0.0000**
Idiap (Cropped-DB)	0.0000	100.00	50.00	0.0000	1.0000	**0.5000**

**Table 12:** Detection errors of our proposed method with and without applying the transfer learning technique using Alex network architecture in Table 2 (unit: %).

Database	Without Transfer Learning				With Transfer Learning			
	SVM Kernel (No. PC)	APCER	BPCER	ACER	SVM Kernel (No. PC)	APCER	BPCER	ACER
ISPR-DB	Polynomial Kernel (No. PC = 60)	1.1875	1.1725	1.1800	Linear Kernel (No. PC = 50)	0.0313	0.0310	**0.0311**
Idiap (Full-DB)	RBF Kernel (No. PC = 50)	0.0000	0.0000	0.0000	RBF Kernel (No. PC = 95)	0.0000	0.0000	0.0000
Idiap (Cropped-DB)	RBF Kernel (No. PC = 150)	1.0000	1.0000	1.0000	Linear Kernel (No. PC = 100)	0.0000	0.0000	**0.0000**

**Table 13:** Detection errors of our proposed method with and without applying the transfer learning technique using VGG-16 network architecture in Table 4 (unit: %).

Database	Without Transfer Learning				With Transfer Learning			
	SVM Kernel (No. PC)	APCER	BPCER	ACER	SVM Kernel (No. PC)	APCER	BPCER	ACER
ISPR-DB	RBF Kernel (No. PC = 200)	2.8438	2.8550	2.8494	Linear Kernel (No. PC = 70)	0.0313	0.0310	**0.0311**
Idiap (Full-DB)	RBF Kernel (No. PC = 70)	0.0000	0.0000	0.0000	Linear Kernel (No. PC = 60)	0.0000	0.0000	**0.0000**
Idiap (Cropped-DB)	RBF Kernel (No. PC = 105)	1.0000	1.0000	1.0000	RBF Kernel (No. PC = 95)	0.0000	0.0000	0.0000

**Table 14:** Detection errors of our proposed method using Alex network architecture in Table 2 on sub-databases of ISPR-DB based on the printing materials (unit: %).

Database	CNN-Based Method with Transfer Learning			Our Proposed Method			
	APCER	BPCER	ACER	SVM Kernel (No. PC)	APCER	BPCER	ACER
Printed on A4 Paper (ISPR-DB1)	0.1875	0.1855	**0.1865**	Polynomial Kernel (No. PC = 110)	0.0313	0.0465	**0.0389**
Printed on MAT Paper (ISPR-DB2)	0.0625	0.0930	**0.0778**	Polynomial Kernel (No. PC = 105)	0.0000	0.0000	**0.0000**
Printed on OHP Film (ISPR-DB3)	0.3750	0.3700	**0.3725**	Polynomial Kernel (No. PC = 90)	0.0938	0.0930	**0.0934**

**Table 15:** Detection errors of our proposed method using Alex network architecture in Table 2 on sub-databases of ISPR-DB based on printing resolutions (unit: %).

Database	CNN-Based Method with Transfer Learning			Our Proposed Method			
	APCER	BPCER	ACER	SVM Kernel (No. PC)	APCER	BPCER	ACER
Printed Using 300 DPI Resolution Printer (ISPR-DB4)	0.1250	0.1390	**0.1320**	Linear Kernel (No. PC = 90)	0.0313	0.0465	**0.0389**
Printed Using 1200 DPI Resolution Printer (ISPR-DB5)	0.0000	0.0000	**0.0000**	Linear Kernel (No. PC = 60)	0.0000	0.0000	**0.0000**
Printed Using 2400 DPI Resolution Printer (ISPR-DB6)	0.2813	0.2775	**0.2794**	Polynomial Kernel (No. PC = 135)	0.0625	0.0930	**0.0778**

**Table 16:** Detection errors of our proposed method using VGG-16 network architecture in Table 4 on sub-databases of ISPR-DB based on the printing materials (unit: %).

Database	CNN-Based Method with Transfer Learning			Our Proposed Method			
	APCER	BPCER	ACER	SVM Kernel (No. PC)	APCER	BPCER	ACER
Printed on A4 Paper (ISPR-DB1)	0.0313	0.0465	**0.0389**	Polynomial Kernel (No. PC = 50)	0.0313	0.0465	**0.0389**
Printed on MAT Paper (ISPR-DB2)	0.0000	0.0000	**0.0000**	Polynomial Kernel (No. PC = 70)	0.0000	0.0000	**0.0000**
Printed on OHP Film (ISPR-DB3)	0.1250	0.1390	**0.1320**	Polynomial Kernel (No. PC = 90)	0.0000	0.0000	**0.0000**

**Table 17:** Detection errors of our proposed method using VGG-16 network architecture in Table 4 on sub-databases of ISPR-DB based on the printing resolution (unit: %).

Database	CNN-Based Method with Transfer Learning			Our Proposed Method			
	APCER	BPCER	ACER	SVM Kernel (No. PC)	APCER	BPCER	ACER
Printed Using 300 DPI Resolution Printer (ISPR-DB4)	0.0313	0.0465	**0.0389**	RBF Kernel (No. PC = 145)	0.0000	0.0000	**0.0000**
Printed Using 1200 DPI Resolution Printer (ISPR-DB5)	0.0000	0.0000	**0.0000**	Linear Kernel (No. PC = 195)	0.0000	0.0000	**0.0000**
Printed Using 2400 DPI Resolution Printer (ISPR-DB6)	0.0625	0.0465	**0.0545**	RBF Kernel (No. PC = 140)	0.0313	0.0465	**0.0389**

**Table 18:** Detection errors of CNN-based method and our proposed method using customized Alex network in Table 3 (unit: %).

Database	CNN-Based Method with Transfer Learning			Our Proposed Method			
	APCER	BPCER	ACER	SVM Kernel (No. PC)	APCER	BPCER	ACER
ISPR-DB	0.6563	0.6640	0.6601	RBF Kernel (No. PC = 70)	0.2500	0.2625	**0.2563**
Idiap Full-DB	0.0000	0.0000	0.0000	Linear Kernel (No. PC = 75)	0.0000	0.0000	**0.0000**
Idiap Cropped-DB	0.0000	0.0000	0.0000	Linear Kernel (No. PC = 55)	0.0000	0.0000	**0.0000**

**Table 19:** Detection errors of CNN-based method and our proposed method using customized VGG-16 network in Table 5 (unit: %).

Database	CNN-Based Method with Transfer Learning			Our Proposed Method			
	APCER	BPCER	ACER	SVM Kernel (No. PC)	APCER	BPCER	ACER
ISPR-DB	0.2500	0.2780	0.2640	Linear Kernel (No. PC = 105)	0.2188	0.2160	**0.2174**
Idiap Full-DB	0.0000	0.5000	0.2500	Linear Kernel (No. PC = 90)	0.0000	0.0000	**0.0000**
Idiap Cropped-DB	1.0000	1.0000	1.0000	Linear Kernel (No. PC = 80)	0.0000	0.0000	**0.0000**

**Table 20:** Detection errors of our proposed method using customized Alex network and VGG-16 network on sub-databases of ISPR-DB database (unit: %).

Database	Using Customized Alex Network				Using Customized VGG-16 Network			
	SVM Kernel	APCER	BPCER	ACER	SVM Kernel (No. PC)	APCER	BPCER	ACER
Printed on A4 Paper (ISPR-DB1)	RBF Kernel (No. PC = 75)	0.3438	0.3240	**0.3339**	Polynomial Kernel (No. PC = 115)	0.2188	0.2315	**0.2251**
Printed on MAT Paper (ISPR-DB2)	Linear Kernel (No. PC = 120)	0.1563	0.1855	**0.1709**	Linear Kernel (No. PC = 75)	0.0313	0.0465	**0.0389**
Printed on OHP Film (ISPR-DB3)	Polynomial Kernel (No. PC = 120)	0.2188	0.2315	**0.2251**	Linear Kernel (No. PC = 50)	0.5625	0.5560	**0.5593**
Printed Using 300 DPI Resolution Printer (ISPR-DB4)	Linear Kernel (No. PC = 55)	0.3438	0.3705	**0.3571**	Linear Kernel (No. PC = 75)	0.2188	0.2315	**0.2251**
Printed Using 1200 DPI Resolution Printer (ISPR-DB5)	Linear Kernel (No. PC = 120)	0.0000	0.0000	**0.0000**	Linear Kernel (No. PC = 200)	0.0313	0.0465	**0.0389**
Printed Using 2400 DPI Resolution Printer (ISPR-DB6)	Polynomial Kernel (No. PC = 120)	0.5938	0.6015	**0.5976**	Linear Kernel (No. PC = 60)	0.0938	0.0925	**0.0931**

**Table 21:** Comparison of PAD errors of our proposed method and various methods using ISPR and its sub-databases (unit: %).

Method	Printed Using 300 DPI Resolution Printer (ISPR-DB4)	Printed Using 300 DPI Resolution Printer (ISPR-DB5)	Printed Using 300 DPI Resolution Printer (ISPR-DB6)	Entire ISPR Database (ISPR-DB)
FFT + HW + DW [24]	2.5460	1.4760	3.9310	2.8740
CNN-based Method Using Alex network Architecture (Without PCA and SVM)	0.1320	0.0000	0.2794	0.1940
CNN-based Method using VGG-16 network Architecture (Without PCA and SVM)	0.0389	0.0000	0.0545	0.0620
**Our Proposed Method** (With PCA and SVM)	**0.0000**	**0.0000**	**0.0389**	**0.0311**

**Table 22:** Comparison of PAD errors using our proposed method and various methods using Idiap Full-DB and Idiap Cropped-DB databases (unit: %).

Database	Method	APCER	BPCER	ACER
Idiap Full-DB	Baseline [23]	0.00	0.00	0.00
	GUC [23]	0.00	8.00	4.00
	B-Lab [23]	0.00	0.00	0.00
	GRIP-PRIAMUS [23]	0.00	0.00	0.00
	CNN-based Method Using Alex network Architecture (Without PCA and SVM)	0.00	0.00	0.00
	CNN-based Method using VGG-16 network Architecture (Without PCA and SVM)	0.00	0.00	0.00
	**Our Proposed Method** (With PCA and SVM)	**0.00**	**0.00**	**0.00**
Idiap Cropped-DB	Baseline [23]	11.00	30.00	20.50
	GUC [23]	1.50	4.00	2.75
	B-Lab [23]	0.00	2.50	1.25
	GRIP-PRIAMUS [23]	0.00	0.00	0.00
	CNN-based Method Using Alex network Architecture (Without PCA and SVM)	0.00	0.00	0.00
	CNN-based Method using VGG-16 network Architecture (Without PCA and SVM)	0.00	0.00	0.00
	**Our Proposed Method** (With PCA and SVM)	**0.00**	**0.00**	**0.00**

**Table 23:** Description of the Idiap palm-vein database (PVD) database used in our experiments for PAD for palm-vein recognition system.

Idiap PVD Database		Training Database		Testing Database
		Original Database	Augmented Database	
Session 1	Full Image Protocol	500	24,500 (500 × 49)	500
	Cropped Image Protocol	500	24,500 (500 × 49)	500
Session 2	Full Image Protocol	500	24,500 (500 × 49)	500
	Cropped Image Protocol	500	24,500 (500 × 49)	500

**Table 24:** Detection errors using Idiap PVD database (unit: %).

Idiap PVD Database		Method	APCER	BPCER	ACER
Session 1	Full Protocol	FFT + HW + DW [24]	2.6	3.6	3.1
		CNN-based Method Using Alex network (Without PCA and SVM)	0.2	0.4	0.3
		CNN-based Method Using Alex network (Without PCA and SVM)	0.0	0.0	0.0
		**Our Proposed Method** (With PCA and SVM)	0.0	0.0	**0.0**
	Cropped Protocol	FFT + HW + DW [24]	4.0	3.2	3.6
		CNN-based Method Using Alex network (Without PCA and SVM)	2.2	3.0	2.6
		CNN-based Method Using Alex network (Without PCA and SVM)	0.2	0.4	0.3
		**Our Proposed Method** (With PCA and SVM)	0.0	0.0	**0.0**
Session 2	Full Protocol	FFT + HW + DW [24]	3.4	1.8	2.6
		CNN-based Method Using Alex network (Without PCA and SVM)	0.2	2.0	1.1
		CNN-based Method Using Alex network (Without PCA and SVM)	0.0	0.0	0.0
		**Our Proposed Method** (With PCA and SVM)	0.0	0.0	0.0
	Cropped Protocol	FFT + HW + DW [24]	5.8	4.4	5.1
		CNN-based Method Using Alex network (Without PCA and SVM)	2.2	2.2	2.2
		CNN-based Method Using Alex network (Without PCA and SVM)	0.2	0.2	0.2
		**Our Proposed Method** (With PCA and SVM)	0.0	0.0	**0.0**
